# LLM-impersonated debate contributions are more authentic, relevant and coherent than their original: A representative study using BBC1’s Question Time

**DOI:** 10.1371/journal.pone.0347757

**Published:** 2026-07-01

**Authors:** Steffen Herbold, Alexander Trautsch, Zlata Kikteva, Annette Hautli-Janisz

**Affiliations:** Department of Computer Science and Mathematics, University of Passau, Passau, Germany; A Sharqiyah University, OMAN

## Abstract

Generative AI has the potential to pollute the public information sphere with made-up content, posing a significant threat to the cohesion of societies at large. This paper offers the first large-scale and systematic study of how authentic, relevant and coherent impersonated content from Large Language Models (LLMs) is perceived by the general public. Based on a cross-section of British society, we show that LLM-generated responses to questions drawn from a broadcast political debate programme in the UK are judged to be more authentic and relevant than the original responses given by the panel members who were impersonated. We also show that stylistic differences do not influence these judgments, meaning that the distinction of original and generated content is challenging for the general public. Taken together, this means that LLMs can be made to deceive the public regarding the nature of statements in the political domain, with the consequence that there is a dire need to inform the general public of the potential harm this can have on society.

## Introduction

Modern Generative Artificial Intelligence models (GenAI) like GPT [1], Claude [2], and Gemini [3] have been shown to generate high-quality textual content including source code [4], persuasive student essays [5], and legal analysis [6]. They also seem (at least partly) to be able to role-play [7], to mimic the linguistic pattern of authors [8], to generate content that reflects general political identity [9,10] and to assume the role of a domain expert who responds accordingly [11]. Whereas developments like these are already questionable in terms of moral and ethical responsibility, Large Language Models (LLMs) influencing the political opinion of humans [12], purporting political bias [13] and successfully generating targeted persuasive communication [14,15] clearly signals that the technological advancement is reaching a critical stage where harm to societies is only a prompt away. This is solidly supported by the findings of this paper.

In our study we condition an LLM in such a way that it impersonates well-known political and societal personalities in a political talk show on UK national television. Specifically, the LLM is tasked to respond to the audience questions in the show as an impersonation of the original speakers on the show. Based on a representative cross-section of British society (n = 948), we study how UK citizens rate the actual response of the person in comparison to the impersonated response along three axes: authenticity (the likelihood that the impersonated response comes from the actual person), coherence (the logical flow of the response), and relevance (the extent to which the response is relevant to the question). We also compare how content and linguistic properties differ between original and impersonated statements and elicit the openness of citizens to using AI technology for generating contribution to public debates. These three lines of research answer the following research questions:

**RQ1:** To what extent do UK citizens rate the authenticity, coherence, and relevance of impersonated debate responses differently from actual debate responses by that person?
**RQ2:** To what extent does a difference in content and linguistic style between actual and impersonated debate responses impact their authenticity ratings?
**RQ3:** What is the general public’s view on using AI in public debates and is this view affected by exposure to technology?

The data underlying our study originates from 30 episodes of BBC1’s Question Time, one of the most viewed political debate programmes in the UK, which were broadcast from 2020 to 2022 [16]. The survey participants are asked to (i) attribute both actual and impersonated responses to public persons; (ii) evaluate how coherent and relevant both actual and impersonated responses are; and (iii) express their opinion regarding the use of AI in public debates. The last task is split into two sub-tasks: First, the participants give their opinions on AI unaware of the source of the material they just rated (actual vs. impersonated). In the next step, they are shown the source of their rated content, and they express their opinion on the technology again.

## Materials and methods

### Data

#### Original debate content.

The original debate data is from ‘Question Time’ (QT), one of the most-viewed political talk shows on UK television. QT30 [16] is a collection of 30 episodes of QT aired on BBC1 between June 2020 and November 2021, currently the largest dataset of analysed broadcast political debate. QT features a moderated panel format, where well-known members of politics and society sit on a panel and respond to questions from the audience on the current topics of the week. The panellists are directed by the moderator and are asked to respond to the questions independent of a prior conversation on the topic and the initial statements by other panellists. The panel members featured in the dataset belong to one of six categories: politicians (50%), business people (16.67%), journalists (14.17%), medical experts (6.67%), writers (5.83%), and other well-known members of UK society (activists, actors, political experts and sports personalities – 6.67%). The use of this data for research is covered by Section 29 (1) of the British Copyright, Designs and Patents Act (CDPA). The data cannot be shared publicly and will be shared with other researchers for the sole use of research upon request (see data availability statement).

From QT30 we manually extract a total of 119 unique questions with 555 responses from 119 different speakers. We discard the responses of seven speakers who do not have a Wikipedia page, a requirement for generating the impersonated debate responses. At the same time, this criterion serves as a filter to determine whether the panel members are well-known personalities in the public sphere. We also discard one response where the corpus data does not contain information about the speaker. This yields a set of 527 valid question/response pairs from 112 different speakers. We randomly drop seven responses to achieve a final count of 520 question/response pairs to facilitate easier sampling. A manual check of the resulting data ensures that the responses are understandable without context and do not refer to other panel members’ previous contributions, as this could affect judgments of authenticity and relevance.

#### Impersonated debate content.

To generate the impersonated responses, we use GPT-4 Turbo [1]. While more recent models, e.g., Opus Claude [2], seem to be slightly better at logical tasks like mathematics, we are not aware of any benchmark where GPT was significantly outscored in tasks that involve common knowledge (as shown, for instance, in [17]). For prompting, we use a complex emulation protocol similar to Bhandarkar et al. [8] with the following prompts:

- System prompt: You are an expert at mimicking different persons in debates. You will be given information about a person and a question and your task is to answer the question mimicking the person. You only answer as the person you are asked to mimic. Do not say the name of the person you are mimicking. Do not introduce yourself. Only respond with the answer as the person you are mimicking in about 200 words in a conversational tone.
- User prompt: Please only answer this question: [QUESTION] as this person: [SPEAKER_WIKIPEDIA]. Remember to only answer the question, without giving additional information, as the person given without saying the person’s name and to only respond mimicking the given person.

The system prompt defines the behaviour we expect from the model, i.e., it is tasked to mimic a well-known person, to be brief in the response to the question and to use a conversational tone. The user prompt defines the task, provides the question and adds the short biography of the speaker that we obtain from the first paragraph of their Wikipedia article (this paragraph provides a summary of the information on their origin, career, party affiliation, political offices etc.) The user prompt also repeats the task for the model.

After receiving the responses, we conduct a manual sanity check to ensure that the impersonated responses adhere to the guidelines given in the prompt, i.e., do not contain the name of the speaker, any information that the response was generated by an LLM or a reason why no response was possible (for instance, due to lack of access to real-time data or for ethical reasons). This check did not flag problematic content, meaning that all responses were used in the subsequent study.

### Study participants

Since the debate content we study originates from one of the most popular British topical debate programs, we recruit a representative sample of British citizens above the age of 18 using the online platform Prolific. The recruitment time frame ran from June 18th until June 26th, 2024. Participants are informed about the purpose of the study, consent to participate and receive a participation fee for compensation which lies above UK minimum wage. We recruit a total of 948 participants, who are distributed randomly across the different tracks that the study comprises, resulting in at least two judgments for each of the 1560 question/response pairs (520 original question/original response pairs, 520 original question/impersonated response pairs, and 520 pairs of original question/original responses with random speaker) in each track.

### Study design

#### Measures and variables.

To evaluate the perception of original and impersonated debate content, we elicit judgments on the following measures:

- *Authenticity*: The likelihood that the response is an actual and real utterance by the speaker in a debate, i.e., the degree to which this utterance could have been contributed by that speaker in a debate. This variable measures the core aspect of our study, i.e., if people believe that a statement is genuine.
- *Coherence*: The logical flow of the response. This variable measures the internal reasoning structure of the response.
- *Relevance*: The extent to which the response addresses the question. This variable measures if the response stays on topic and conveys relevant information.
- *Content*: The extent to which the overall meaning of original and impersonated response is identical. This variable allows us to understand if LLM-generated responses differ from the actual responses.
- *Confidence*: The confidence in judging whether the response was given by a specific speaker. This variable is used as a control variable to understand if the certainty in judging debate content is affected by whether it is original or impersonated.
- *Familiarity*: The knowledge on a panel member based on their previous public appearances. This variable is used as a control variable to understand if familiarity with a speaker has an impact on the authenticity judgments.

For all measures we use a five-point Likert scale (for instance, ‘not authentic’ to ‘very authentic’) such that the middle point of the scale is neutral. The supplementary material contains the full description of the measures. The judgments are collected using an online survey, the design of which we present in the following.

#### Study procedure.

Our custom-made online survey starts with the collection of demographic data on the participants, i.e., their age, gender, country of residence within the United Kingdom, and political preference. At this stage, the participants are only informed that the debate questions and responses are taken from the BBC1 show ‘Question Time’. They are not aware that some of the responses are generated by an LLM (this information is disclosed only at a later stage of the survey). This deceptive design prompts participants to believe they are judging actual debate content.

The participants are then randomly sampled into three tracks. *Track1* measures the perception of the authenticity, coherence, and relevance of a single debate response given a question. The participants are shown the question, one response, and the name of the speaker. The response is either the original response by the speaker or an LLM-generated, impersonated response, as described above. *Track2* augments this setting by showing original and impersonated response side-by-side: the participants see the question, the name of the speaker and both responses at the same time. Their task is to compare the responses in terms of authenticity, coherence, and relevance to the question. We remove position bias by randomly positioning original and impersonated responses on the left or the right side of the page. Track2 also measures whether the content of the impersonated responses is the same as that of the actual responses.

With *Track3* we try to understand better the factors that lead to differences in authenticity: Here, participants are shown a question, a response, the name of the speaker, and the speaker’s short biography. The biography is the same one that we provide to the LLM as part of the user prompt. For statistical reasons, we create three populations of participants. The first population sees the question, the actual speaker, their biography and the actual response. The second population sees the question, the actual speaker, their biography and the impersonated response. And the third population sees the question, the actual response from the actual speaker, but a randomly selected different public person from our data set, plus that person’s biography. All participants are asked to judge the authenticity of the responses, to rate their confidence in the judgment and to rate their familiarity with the speaker.

Once the participants complete their respective tracks, they participate in an exit poll. Here we ask questions regarding their familiarity with AI and chatbots, their opinion on the use of AI in public debates, and the perceived need for transparency and regulation in this setting (see more details in the supplemental material S1 Appendix). Only after the exit poll is completed, we reveal to the participants which of their judged debate responses were generated by an LLM, together with their ratings. In light of this new information, we repeat the exit poll for all participants. This allows us to see whether more insight into the quality of the generated responses affects their opinions regarding the use of AI in public debates. As a last step, the participants are then invited to provide an (optional) free-text comment regarding their answers in the exit poll.

Overall, each participant judges eight different responses. For Track1 and Track3 this means that each participant judges eight question/response pairs (we use rejection sampling to ensure each question/speaker pair only appears once, i.e., it is not possible for a participant to judge both the actual and the impersonated responses from a speaker to a question). For Track2 this means that each participant judges four pairs of question/original plus impersonated response. Additional details about the survey, including the exact wording of the questions, are provided in supplemental material S1 Appendix.

### Stylistic comparison

We also measure stylistic differences between the original and the impersonated responses by comparing discourse-related linguistic patterns. This allows us (1) to understand if the responses share properties on the linguistic surface and (2) whether the language is related to human judgments in terms of authenticity, coherence, and relevance. The study is based on the following linguistic properties:

- *Syntactic complexity*: Syntactic complexity in terms of the mean number of conjuncts, clausal modifiers of nouns, adverbial clause modifiers, clausal complements, clausal subjects and parataxis per sentence as an approximation of language complexity. [18]
- *Nominalisations*: The number of nominalisations per sentence as an approximation of the of abstractness of the language.
- *Modals*: The number of modal constructions (e.g., ‘definitely’, ‘potentially’) per sentence as a signal of the stance of the speakers towards their utterances. [19]
- *Discourse markers*: The number of discourse markers (e.g., ‘first’, ‘moreover’) per sentence as an approximation of the coherence of text and the use of explicit argumentative structure. [20]
- *Epistemic markers*: The number of epistemic markers (e.g., ‘I think’, ‘in my opinion’) as an indication of the commitment of a speaker to the message they convey.
- *Lexical diversity*: Lexical diversity measured with MTLD [21] as an approximation of the diversity of the used vocabulary.
- *Lexical overlap*: The percentage of words in the question (excluding stop words) that also appear in the response as an approximation of the influence of the question on the response.

These stylistic features are automatically extracted from the original and the generated responses using a combination of rule-based, stochastic and neural models for natural language processing. Modals, discourse markers, epistemic markers, nominalisations, and the lexical markers are normalised by the number of sentences within a response.

### Statistical analysis

The inter-rater reliability between the two judgments for authenticity, coherence, relevance and content is measured with Cronbach’s α [22]. Additionally, we report pair-wise differences between the judgments to quantify the disagreement between participants. We exclude confidence and familiarity because we cannot expect agreement regarding a subjective self-reflection. For the subsequent statistical analysis, we map the Likert scales to the integers [−2, −1, 0, 1, 2] and compute the average rating between the two judgments for the same data point. Since the variables from our survey are based on Likert scales, we use non-parametric rank-based statistical tests.

In Track1 we assess the difference in authenticity, coherence, and relevance between the original responses and the impersonated responses. The track has a between-subjects design (i.e., actual and impersonated responses are rated by different participants) with data that is paired by the question and the speaker. Consequently, we use a two-sided Wilcoxon signed rank test [23] to determine if the difference between both populations (actual versus impersonated responses) is significant.

Track2 uses a within-subjects design (i.e., one participant judges both the actual and the impersonated response). We conduct a two-sided one-sample Wilcoxon signed rank test to determine if the judgments regarding authenticity, coherence, relevance, and content are significantly different from zero. For authenticity, coherence, and relevance, a significant tendency towards negative values means that the participants favour the original responses; a significant tendency towards positive values means that the generated, impersonated responses are favoured. Regarding content, a significant positive value means that the content between original and impersonated responses is similar; a negative value means that the impersonated content is different from the actual content by the speaker. We post-process the data such that the original response is always on the left and the impersonated response is always on the right.

With the data from Track3 we assess if the authenticity and the confidence in the rating depend on whether the speaker is real, random, or impersonated. This results in three populations. The track has a between-subjects design where the populations are paired by the question and the actual speaker. To elicit whether there is any difference between the three populations, we use a Friedman test [24] with a Bonferroni-Dunn post-hoc test based on pair-wise two-sided Wilcoxon signed rank tests. This determines which differences between pairs are significant. Additionally, we use familiarity judgments to understand how this affects authenticity. For this, we conduct a subgroup analysis where we split the ratings into those where the familiarity is less than 0 (i.e., ratings where the participants are not/to a limited extent/fairly familiar with the speaker) and judgments with a familiarity greater than or equal to 0 (speakers are somewhat familiar/familiar with the speaker). For the latter subgroup, we do not have paired data anymore, because we have independent raters for the three populations. For instance, the raters for the responses attributed to the actual speakers may be familiar with different speakers than the raters for the impersonated responses, leading to different subgroups. We therefore use a Kruskal-Wallis test [25] with Bonferroni-Dunn post-hoc tests based on pair-wise two-sided Wilcoxon–Mann–Whitney tests [26].

The statistical analysis of the linguistic surface markers is similar to the analysis of Track1 since we also have two populations for each linguistic marker (actual versus impersonated responses). Since the data from the linguistic markers does not follow a normal distribution (visual analysis of the distribution in Fig 5 shows, for instance, long tails), we also use non-parametric tests, namely two-sided Wilcoxon signed rank tests to determine if differences for each of the seven linguistic markers are significant.

**Fig 1:**
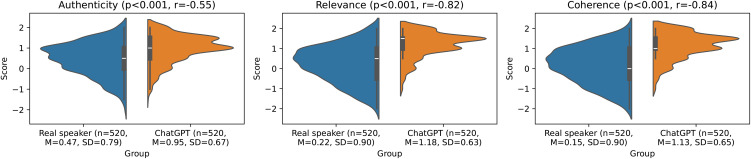
Judgments when a debate question, the name of the speaker, and either the GPT-generated or the actual response by the actual speaker are shown. Violins show a kernel density estimation of the probability distribution, the miniature box-plots depict the median, upper and lower quartiles, and the whiskers the largest/smallest value observed within 1.5 times the interquartile range of the upper/lower quartile. The statistical markers reported are the p-value of two-sided Wilcoxon signed rank tests, the effect size *r*, the sample sizes *n*, mean values *M* and standard deviations *SD*.

**Fig 2:**
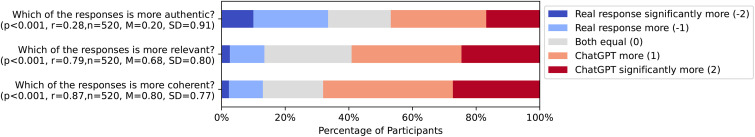
Judgments when a debate question, the name of the speaker, and both the actual and GPT-generated responses are shown side-by-side. The stacked bar chart reports the percentages of the ratings that we observed. The statistical markers reported are the p-value of a two-sided one-sample Wilcoxon signed rank tests for a difference from zero, the effect size *r*, the sample sizes *n*, mean values *M* and standard deviations *SD*.

**Fig 3:**
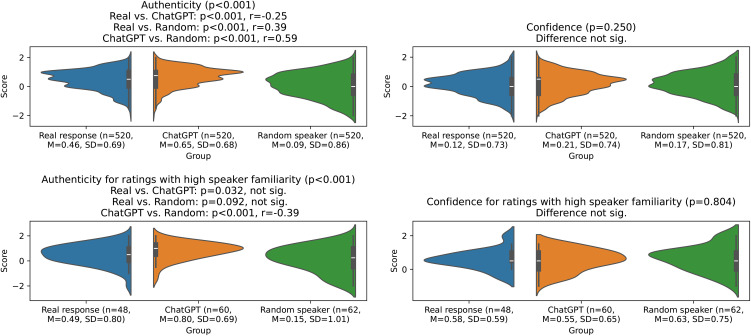
Judgments when a debate question with either the response and biography from the actual speaker, the GPT-generated response and the biography of the actual speaker, or the response from the actual speaker but the name and biography of a random public person are shown. Violins show a kernel density estimation of the probability distribution, the miniature box-plots depict the median, upper and lower quartiles, and the whiskers the largest/smallest value observed within 1.5 times the interquartile range of the upper/lower quartile. The statistical markers reported are the p-value of the omnibus test for differences and pair-wise Bonferroni-Dunn correct two-sided post-hoc tests, the effect size *r*, the sample sizes *n*, mean values *M* and standard deviations *SD*.

**Fig 4:**
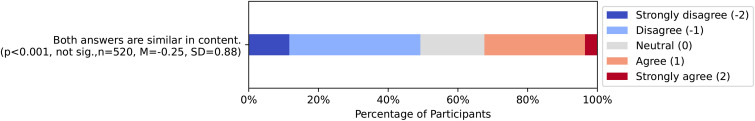
Judgments whether the content of the actual response and the GPT-generated response are the same. The actual and impersonated responses are shown side-by-side. The stacked bar chart reports the percentages of the ratings that we observed. The statistical markers reported are the p-value of a two-sided one-sample Wilcoxon signed rank tests for a difference from zero, the effect size *r*, the sample sizes *n*, mean values *M* and standard deviations *SD*.

Thus, we conduct three statistical tests with the data of Track1, four with the data of Track2, three with the data of Track3 and seven statistical tests for the linguistic markers, i.e., a total of 17 tests. We use a conservative approach based on Bonferroni correction [27] to account for multiple tests and consider results as significant if the p-value of a test is less than $\alpha =\frac{0.05}{17}\approx 0.003$. Based on the large size of our populations with 520 question/response pairs and assuming that we observe differences of 0.5 points (i.e., half a step on the Likert scales), we compute the expected statistical power as β=1. Consequently, differences of 0.5 or larger in judgment should always be picked up by our tests and if there are no differences in judgments, there is only a 5% chance that we find a difference that is not there.

We report the arithmetic mean (M) and standard deviation (SD) as statistical markers for the populations. While our data is not perfectly normal, it also does not have severe outliers or multimodalities, so we prefer the clear interpretation of the arithmetic mean (M) and standard deviation (SD) to report statistical markers for populations. We use the biserial rank correlation *r* [28] to measure the effect size. Violin plots visualise the distribution of the data based on a kernel density estimation of the underlying probability distribution. The violins include miniature box plots that depict the median, upper and lower quartiles and the whiskers defined as the largest/smallest observed value at most 1.5 times the inter-quartile range away from the upper/lower quartile. Additionally, we use stacked bar charts to depict ratios of Likert scale items, where appropriate.

Supplemental material S2 Appendix provides details regarding the results, e.g., the demographic information of the participants. Supplemental material S3 Appendix reports on the results of additional quantitative analysis of possible confounding factors, i.e., randomisation of speakers assigned in Track3, the lengths of responses, and the influence of spelling errors. The results for all these factors are negative, i.e., it is highly unlikely that these aspects serve as alternative explanations of our findings.

The statistical analysis of the data is mostly implemented in Python. We use pandas 2.2.2 and numpy 1.26.4 for processing the data, pingouin 0.5.4 for the calculation of Cronbach’s α, effect sizes *r* and pair-wise tests, scipy 1.13.0 for the omnibus tests, and seaborn 0.13.2 for the generation of plots. We compute the statistical power with the R package mkpower 0.9.

### Qualitative analysis

The qualitative analysis in this study is two-fold: On the one hand, we code the free-text answers from the exit poll of the survey using inductive coding [29] and have one author assign one or more codes to each answer. The codes are aimed to capture the intent of the free-text answer, e.g., convey the reason for changes in the exit poll or observations regarding the impersonated content that the participants found striking (details on the codes are in supplemental material). This is initially done for twenty answers, at which point the coding is checked by and discussed with a second author, resulting in an agreed-upon coding taxonomy. The first author then continues to code the remainder of the data. Upon completion of this coding, the second author again checks all codes and discusses the coding to achieve agreement in the same manner as for the initial set of codes. We then conduct one round of axial coding [30] to group related codes into categories. Same as above, the axial coding is initially conducted by one author, then checked by and discussed with a second author to achieve agreement. We do not report inter-rater agreements for this data, because all data is fully checked by two authors and all disagreements are discussed and resolved, which results in final coding on which both annotators perfectly agree.

On the other hand, we conduct a qualitative analysis to better understand the human judgments regarding the differences in content between actual and impersonated responses, with a specific focus on whether the stance between actual and impersonated responses differs. For this, we randomly sample 50 pairs of actual and generated responses that were judged to be different in content by the study participants. We use deductive coding to determine if (a) the two responses have the same stance, i.e., arrive at the same conclusion, argue for the same points or take the same side, (b) express a different stance, i.e., draw different conclusions or argue for another position, or (c) whether this distinction is not applicable, e.g., because the question does not require the hearers to take a stance. Additionally, we determine for each response whether it addresses the question. Similar to the coding procedure above, the coding was first done by the one author and then checked by a second author. Disagreement between the two annotators was minimal and adjudicated to achieve agreement.

### Ethics statement

Our study used human subjects as participants in a survey. The ethics committee of the University of Passau approved this study (ref. III/Herbold.I-07.5095/240229). The study makes use of an online survey that obtained informed written consent prior to participation in the study.

## Results

### Impersonated responses are perceived as more authentic, coherent and relevant

The results clearly show that LLM-generated, impersonated content is judged as more authentic, coherent, and relevant than the actual debate responses. When the participants only see one question and its response (either actual or impersonated) (see Fig 1), we observe a significant difference across all three dimensions with a large effect size for authenticity (*r* = −0.55), relevance (*r* = −0.82) and coherence (*r* = −0.84) in the responses. When the participants directly compare an impersonated response with an original response along these dimensions (see Fig 2), the results are supported: The effect sizes for relevance (*r* = 0.79) and coherence (*r* = 0.87) remain large, but the difference in authenticity decreases to a weak effect (*r* = 0.28), so the gap between impersonated responses and actual responses is smaller in this setting (but it is still statistically significant). If the participants see the biographies of the speakers during rating (see Fig 3, top-left), we observe a similar effect size for the authenticity when comparing actual and generated responses than when the questions are shown side-by-side (*r* = 0.25). This means that the authenticity of the impersonated response is still higher than that of the actual response, even if biographies are provided.

An important control in our study is whether the debate content is just generally assumed to be authentic, instead of only when the response matches the common knowledge that the public has about the speaker. The data in Fig 3 shows that when deliberately assigning an actual response to a randomly picked (wrong) speaker, the authenticity is significantly lower compared to when the response is assigned to the actual speaker with the actual response (*r* = 0.39) or the actual speaker with the impersonated response (*r* = 0.59). When we take into account the confidence of the participants in their rating, we do not find any significant differences in the certainty of attributing original or impersonated responses to the actual speaker, or attributing an actual response to a random speaker. If we only consider a subgroup of data where the participants are highly familiar with the speakers, this again neither affects the authenticity nor the confidence: While the significance tests for differences between actual and impersonated responses, as well as actual speakers versus random speakers, are not significant anymore, the distributions are almost exactly the same as for the full dataset. This indicates that there is no shift in distributions, but rather the effects are too small to be detected with the smaller subgroups.

### Original content is different from impersonated content

If the authenticity is rated high, but the content of the original response differs from the content of the impersonated response, i.e., the impersonated content is not in line with the actual statements of the person, we are faced with a situation where GenAI can be employed for targeted misinformation about the speaker’s point of view. Our results show that a significant majority of actual responses are judged to be different in content from the impersonated counterparts, though the spread in the distribution is fairly large (see Fig 4). About half of the responses are considered to be dissimilar in comparison to only about one-third of responses that are considered similar. We observe no notable pattern or correlation between the similarity of the content and the authenticity of the responses (ρ=−0.16).

For the qualitative analysis of the differences, we sample 50 of the total 255 pairs of actual and generated responses for which the content is judged as being dissimilar. Table 1 summarizes the results of the analysis. Three categories emerge: The first category comprises cases where the differences in content are about how arguments are presented, but where both the actual and the generated response arrive at the same conclusion, while also addressing the question (*n* = 16 for responses with a stance, *n* = 5 without a stance, 42%). The second category comprises cases where only the actual speaker (*n* = 2, 4%) or only GPT (*n* = 14, 28%) responds to the question and the other source dodges the question. We do not annotate these instances as exhibiting a difference in stance – one side does not elaborate on the topic of the question and therefore does not communicate a stance towards it at all, so the identification of the difference in stance is not applicable. Overall, this means in 32% of cases, the question is dodged by one source. The third (and most important category) captures cases where the stance between the actual response and the generated response is notably different (*n* = 13, 26%). If we extrapolated this to the full dataset, we would expect that around 13% of generated responses communicate a different stance than the actual response.

**Table 1:** Results of qualitative analysis of the differences in content.

Content similarity	Count
Same stance, both address question	16
Stance not required, only generated response addresses question	14
Different stance, both address question	13
Stance not required, both address question	5
Stance not required, only actual response addresses question	2

### Human judgment is reliable

We measure the inter-rater reliability with Cronbach’s α [22] for the two judgments on authenticity, coherence, relevance, and content that we get for each question/response pair. The ratings are based on a five-point Likert scale. Additionally, we report the pair-wise differences between the two participants to understand which disagreements our participants have. We exclude confidence and familiarity because we cannot expect agreement regarding a subjective self-reflection. For the subsequent statistical analysis, we map the Likert scales to the integers [−2, −1, 0, 1, 2] and compute the average rating between the two judgments for the same data point. Since the measures from our survey are based on Likert scales, we use non-parametric rank-based statistical tests.

In all variables, we observe an overall modest agreement when measured with Cronbach’s α, with values of at most α=0.55. We analyse the data to understand which combinations of different judgments we observed and find that regarding authenticity (across all variants) there are relatively few polar differences, i.e., one participant rating an item as authentic and the other as not authentic. For relevance, coherence and content the differences are in how positive a judgment is, with small differences of a single point (e.g., ‘neutral’ instead of ‘agreement’), again showing that while the absolute ratings have some variance, the tendency regarding the judgment is typically the same for both participants. Overall, the tendency towards positive or negative judgments about a variable is fairly consistent, especially given our large sample size which is a representative cross-section of the British society.

We note that our method neither allows us to observe participants directly nor measures underlying aspects regarding their capabilities (e.g., their reading comprehension). In principle, if participants with different capabilities are not evenly assigned to tracks, this could lead to unobserved confounding effects. However, given our large sample size, a strong influence of such confounding effects is unlikely.

### Linguistic style is different

**Fig 5:**
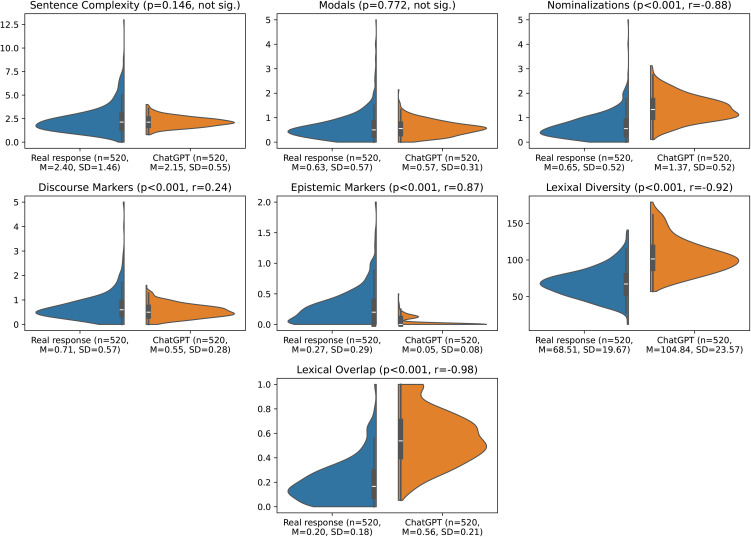
Linguistic surface of actual debate responses versus impersonated debate responses. Violins show a kernel density estimation of the probability distribution, the miniature box-plots depict the median, upper and lower quartiles, and the whiskers the largest/smallest value observed within 1.5 times the interquartile range of the upper/lower quartile. The statistical markers reported are the p-value of two-sided Wilcoxon signed rank tests, the effect size *r*, the sample sizes *n*, mean values *M* and standard deviations *SD*.

To provide a perspective different from the human evaluation, we augment our results with a comparison of the linguistic properties of the original and the generated responses (see Fig 5). This experiment yields a number of interesting insights: First, the complexity of the sentences in terms of the number of conjuncts, clausal modifiers, clausal complements, clausal subjects and parataxes as well as the use of modals such as ‘should’ and ‘must’ are not significantly different between the actual responses and the impersonated ones. Secondly, the actual responses contain more discourse markers (e.g., ‘because’, ‘therefore’) than the impersonated responses, even though with a small effect size (*r* = 0.24). The reason for the statistically significant difference is that there is a long tail of actual responses that contain many discourse markers, even though the peak of the distributions is the same for actual and impersonated responses.

Epistemic markers like ‘I think’ are used substantially more often in original responses – they are only rarely found in the impersonated statements, leading to a large effect size (*r* = 0.87). The rarity in generated responses is not surprising, giving that these markers indicate that the speaker has a stance on some issue, which we expect to be strongly the case in a broadcast political debate and much less so in the case of an LLM.

Furthermore, the impersonated responses contain more nominalisations (*r* = −0.88) and have a higher lexical diversity (*r* = −0.92), both with large effect sizes. The overlap between the words from the question and the response is higher for impersonated than actual responses, with a large effect size (*r* = −0.98). In fact, the distribution shows that it is not uncommon for all words from the question to appear in the impersonated responses, while this is only rarely true for the actual responses.

### Public opinion

Public opinion on the use of AI technology for public debates is collected in two rounds in the exit poll: First, the participants respond without prior knowledge of the data source they just rated (actual versus generated). In the second step, the source of the data is revealed and the participants are asked the same questions on the use of AI technology again.

**Fig 6:**
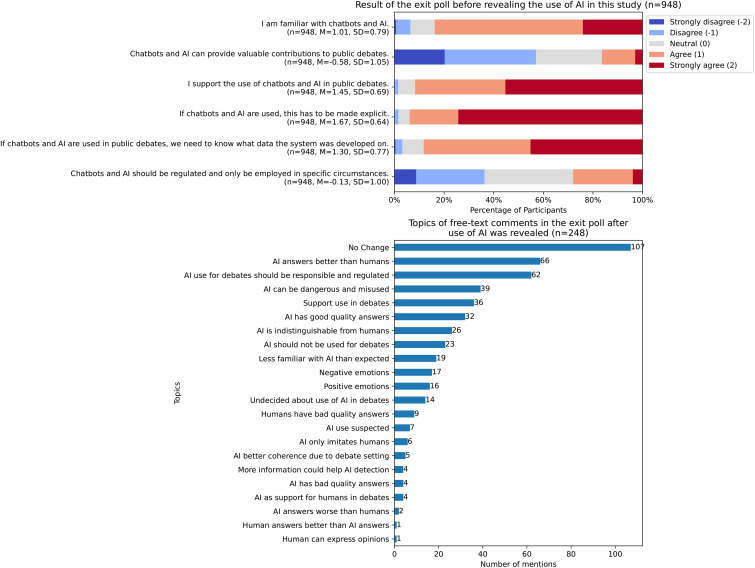
Results of the exit poll on the opinion of the participants. The stacked bar chart reports the percentages of the ratings that we observed. The statistical markers reported are the sample sizes *n*, mean values *M* and standard deviations *SD*. The bar chart depicts the counts for each topic that was addressed in the free-text answers.

The results of our exit poll prior to revealing the use of AI (see Fig 6) paint a clear picture: The participants mostly state that they are familiar with AI. Interestingly, while they mostly believe that AI cannot provide valuable contributions to public debates, they simultaneously claim that they support the use of AI if it is made explicit and if it is known how the system was developed. When it comes to the matter of regulating AI, the participants’ opinions are rather mixed, with roughly equal-sized groups favoring regulation, opposing regulation, and being undecided. After revealing the use of AI, over 90% of the participants do not change their opinion. For those who do change their opinion, we see a clear trend: The participants realize they are less familiar with AI than they thought, but also have a more favorable opinion on the use of AI in debates, while at the same time seeing a bigger need for regulation.

The optional free-text answers (*n* = 248) further corroborate these results. Many participants explicitly note that they did not change their results (*n* = 107). However, the other free-text answers indicate that the changes in opinions are caused by the confrontation with the capabilities of AI through the survey. Participants often mention that the impersonated responses are better than the human responses (*n* = 66) or that the quality of the impersonated responses is higher (*n* = 32). A few participants noted that the high coherence in the impersonated responses made them sceptical, leading them to believe that AI was possibly used in the survey (*n* = 7) and that this advantage over the humans can be explained by the live debate setting where the actual panellists do not have time to carefully prepare their responses (*n* = 5). One participant even notes that this advantage of AI means that AI could be used to train humans for debates. Some participants note that they are not able to distinguish between AI and humans at all (*n* = 26). There are also a few comments noting negative aspects regarding the quality of the generated responses (*n* = 4) or that AI was worse than the humans (*n* = 1), but these are outliers.

Another aspect that is stressed in the comments is the requirement to regulate the use of AI (*n* = 62), especially with respect to transparency. Particularly, many participants (*n* = 39) express concerns regarding the potential for deceptive use of AI in debates and the associated risks, some even note feelings of fear, shock, and worry (*n* = 17). However, some participants express positive emotions like surprise and amazement given the strong capabilities of AI (*n* = 16). When it comes to the use of AI technology in debates, some participants argue that AI’s convincing performance indicates potential for its use in debates (*n* = 36), while others question the general concept of AI debaters (*n* = 23). For instance, participants are uncertain about AI’s ability to represent party opinions and are concerned that involvement of AI will undermine the value of debates as forums for meaningful discussion between people.

## Discussion and conclusion

Our results demonstrate that **modern AI based on LLMs is able to provide high-quality impersonated debate content that is perceived as authentic when attributed to actual people**. We also find indications that people in general rate the impersonated content to be slightly more authentic than the actual human debate responses. In addition, the impersonated responses are judged as more coherent and relevant than actual responses. While the lower coherence can be attributed to the panellists being under scrutiny in a nationally broadcast political debate programme, the higher relevance of the LLM-generated responses indicates that the LLM tends to stay more on-topic than human speakers. Our analysis of responses judged as different in content supports this, as we find that the panel members do not address the question more often than the LLM. While we rule out length and grammatical errors as possible sources for differences in authenticity, we cannot rule out that there are unobserved factors introduced by our experiment design. Interestingly, the authenticity is not negatively affected by the notable differences in the linguistic surface of the responses. GPT clearly has its own unique style defined by a diverse vocabulary and an avoidance of epistemic markers, which is, however, not noticed by our study participants. There does not seem to be a problem with an uncanny valley [31], which would make the participants feel uncomfortable with the impersonated responses.

Even though most of **our participants stated that they are familiar with AI, they did not expect AI to have generated these responses and therefore underestimated the capabilities of modern generative AI**. Being confronted with the AI’s ability to generate convincing debate contributions elicited different reactions from the participants including evidence-driven discussions of the merits of AI, negative emotional responses driven by concerns over the potential for misuse, and positive emotional responses to technological progress demonstrated by the AI’s capabilities. Overall, the participants’ encounter with AI via our survey seemed to increase their appreciation for generative technology in some ways, while also highlighting the need for regulation of such tools when used in a debate context.

When questioned about the merits of AI, the participants expressed a strong belief that AI can be a valuable tool, but they have heterogeneous views on the need for regulation and restrictions on use. However, on the matters of transparency, the public perspective is clear: **Over 85% of participants think that AI use has to be made explicit and that information on how the AI was developed needs to be shared**.

In general, the risks that are implied by our findings are severe. While it has been previously established that LLMs are capable of generating persuasive misinformation [32] and that the automated and human detection of such misinformation is unreliable [33], the results of our study add another layer of concern: We demonstrate that LLMs can generate authentic information by impersonating specific people, meaning that LLM-powered misinformation campaigns can go beyond targeting general topics and societal group, but can be made to target individual people by imitating their contributions to public discourse. Furthermore, the potential for AI to generate responses that do not only successfully imitate a politician, but also push a specific political agenda, warrants a larger-scale further exploration and an assessment of the associated risks. We note that our setting did not study this targeted misinformation, i.e., we did not prescribe the position the LLM should express. Future work needs to study if LLMs are still perceived as authentic when used in such a targeted manner. Since the dissemination of excerpts from political statements via social networks is a common form of political communication [34], it is easy to spread such generated statements at scale. Therefore, content moderation to identify and remove undisclosed AI-generated statements will be crucial [35]. Our own results suggest that a current model [36] can be used for such content moderation (accuracy of 89% on the task of classifying responses into impersonated or actual). However, more sophisticated approaches may be able to fool such detectors [37].

Overall, the implications of our results for the communication of political content are devastating: **Threat actors can easily use LLMs to pollute public information spheres with fake, but authentic-sounding, political statements**, for instance, to sow confusion about the original statement and to invent talking points. If this is further combined with deep fakes that are already known to be able to generate reliable authentic voices and videos of public people [38], the risk for society is enormous.

## Supporting information

S1 Appendix Additional details for the survey. (PDF)

S2 Appendix Additional details for the results. (PDF)

S3 Appendix Additional analysis of alternative explanations for results. (PDF)
