# Supplementary material for: LLM-impersonated debate contributions are more authentic, relevant and coherent than their original: A representative study using BBC1’s Question Time
Source: PLoS One. 2026 Jul 1;21(7):e0347757. doi: 10.1371/journal.pone.0347757 (PMC13322533; doi:10.1371/journal.pone.0347757)
Supplement: S1 Appendix — (PDF) [file pone.0347757.s001.pdf]

S1 Appendix: Additional details for the survey

This supplemental material provides additional details about the survey of British citizens to judge the actual and GPT-generated debate content.

Survey questions and scales

Tables 1, 2, 3, 4 and 5 specify the questions and scales used for our survey.

Table 1. Demographic data collected as part of the survey.

Question	Scale
Age	Age in years
Gender	Male, Female, Other, Prefer not to disclose
Country of Residence	England, Scotland, Wales, Northern Ireland
Politically interested	Yes, No, Prefer not to disclose
Political preference	Conservative Unionist Party, Labour Party, Scottish National Party, Liberal Democrats, Democratic Unionist Party, Sinn Fein, Plaid Cymru, Social Democratic and Labour Party, Alba Party, Green Party of England and Wales, Alliance Party of Northern Ireland, Ulster Unionist Party, Scottish Greens, Traditional Unionist Voice, People Before Profit, No Preference, Prefer not to disclose

Table 2. Questions and Likert scales used for Track1, where a single question, the speaker name, and either the GPT-generated or the actual response were shown.

Question	Scale
Q1.1: The response to the question is authentic.	Strongly disagree, Disagree, Neutral, Agree, Strongly Agree
Q1.2: The response to the question is coherent.	Strongly disagree, Disagree, Neutral, Agree, Strongly Agree
Q1.3: The response to the question is relevant.	Strongly disagree, Disagree, Neutral, Agree, Strongly Agree

Table 3. Questions for the Track2, where original and impersonated response are shown side-by-side.

Question	Scale
Q2.1: Which of the responses is more authentic?	Left is significantly more authentic, Left more authentic, Both equally authentic, Right more authentic, Right significantly more authentic
Q2.2: Which of the responses is more relevant to the question?	Left is significantly more relevant, Left is more relevant, Both equally relevant, Right more relevant, Right significantly more relevant
Q2.3: Which of the responses is more coherent?	Left is significantly more coherent, Left is more coherent, Both equally coherent, Right more coherent, Right significantly more coherent
Q2.4: Both answers are similar in content.	Strongly disagree, Disagree, Neutral, Agree, Strongly Agree

Table 4. Questions and Likert scales used for Track3, where a single question, the speaker name was shown together with the GPT-generated and actual response side-by-side.

Question	Scale
Q3.1: The response to the question came from the speaker described above.	Strongly disagree, Disagree, Neutral, Agree, Strongly Agree
Q3.2: I am confident in my previous judgment.	Strongly disagree, Disagree, Neutral, Agree, Strongly Agree
Q3.3: I am familiar with the speaker described above.	I am not familiar with the speaker, My familiarity with the speaker is limited, I am fairly familiar with the speaker, I am somewhat familiar with the speaker, I am familiar with the speaker

Table 5. Questions and Likert scales used for the exit poll.

Question	Scale
E1: I am familiar with chatbots and AI.	Strongly disagree, Disagree, Neutral, Agree, Strongly Agree
E2: Chatbots and AI can provide valuable contributions to public debates.	Strongly disagree, Disagree, Neutral, Agree, Strongly Agree
E3: I support the use of chatbots and AI in public debates.	Strongly disagree, Disagree, Neutral, Agree, Strongly Agree
E4: If chatbots and AI are used, this has to be made explicit.	Strongly disagree, Disagree, Neutral, Agree, Strongly Agree
E5: If chatbots and AI are used in public debates, we need to know what data the system was developed on.	Strongly disagree, Disagree, Neutral, Agree, Strongly Agree
E6: Chatbots and AI should be regulated and only be employed in specific circumstances.	Strongly disagree, Disagree, Neutral, Agree, Strongly Agree

Codes and categories of free-text answers

Below, you find the list of codes and categories we assigned to the free-text answers.

- *AI as support for humans in debates*: ai_as_tool, ai_use_training_tool
- *AI has bad quality answers*: ai_bad_quality, ai_bad_quality_authentic, ai_bad_quality_confusing_statements, ai_bad_quality_sentences
- *AI better coherence due to debate setting*: ai_better_coherence_expected, ai_better_quality_coherence_expected
- *AI answers better than humans*: ai_better_quality, ai_better_quality_accuracy, ai_better_quality_argumentation, ai_better_quality_articulate, ai_better_quality_authentic, ai_better_quality_authenticity, ai_better_quality_clearer, ai_better_quality_coherence, ai_better_quality_coherent, ai_better_quality_convincingness, ai_better_quality_detailed, ai_better_quality_evidence_based, ai_better_quality_flow, ai_better_quality_fluency, ai_better_quality_grammar, ai_better_quality_honesty, ai_better_quality_informative, ai_better_quality_less_emotion, ai_better_quality_reasoning, ai_better_quality_relevance, ai_better_quality_relevance, ai_better_quality_structure, ai_better_quality_understanding, ai_better_quality_usefulness
- *AI use for debates should be responsible and regulated*: ai_data_source, ai_use_disclosed, ai_use_regulated, ai_use_regulated_limited, ai_use_responsibly, ethical_concerns
- *AI can be dangerous and misused*: ai_fact_correctness, ai_misuse, ai_replace_humans, ai_use_caution, ai_use_danger, ai_use_deceive, ai_use_misuse
- *AI has good quality answers*: ai_good_balanced, ai_good_quality, ai_good_quality_accuracy, ai_good_quality_adapts_to_new_topics, ai_good_quality_authenticity, ai_good_quality_balanced, ai_good_quality_clarity, ai_good_quality_coherence, ai_good_quality_considered, ai_good_quality_convincing, ai_good_quality_convincingness, ai_good_quality_detailed, ai_good_quality_fluency, ai_good_quality_reasoning, ai_good_quality_relevance, ai_good_quality_sentence_structure, ai_quality_good_summarization
- *AI only imitates humans*: ai_use_imitates

- *AI is indistinguishable from humans*: ai_indistinguishable
- *Less familiar with AI than expected*: ai_not_as_familiar
- *AI use suspected*: ai_too_coherent, suspected_ai_involvement
- *AI should not be used for debates*: ai_use_defeats_purpose, ai_use_limited, ai_use_no
- *Undecided about use of AI in debates*: ai_use_maybe, ai_use_unclear, ai_use_undecided, ai_use_undecided'
- *Support use in debates*: ai_use_yes, ai_valuable_contribution, ai_valuable_contribution, ai_valuable_contributions
- *AI answers worse than humans*: ai_worse_quality_authenticity, ai_worse_quality_novelty
- *More information could help AI detection*: awareness_could_help, context_to_distinguish
- *Other*: bad_quality_does_not_matter, change, familiar_with_QT, made_mistake_in_survey, nature_of_ai, no_change, satisfied_with_responses, study_encourages_caution, survey_structure_comment, time_tracker_issue, unfamiliar_with_speakers
- *Negative emotions*: emotion_alarmed, emotion_confusion, emotion_deceived, emotion_dismay, emotion_fear, emotion_shock, emotion_unhappiness, emotion_worry
- *Positive emotions*: emotion_amazement, emotion_fascinated, emotion_impressed, emotion_surprise, emotion_surprised
- *Humans have bad quality answers*: human_quality_bad, human_quality_bad_coherence, people_lie, quality_human_bad
- *Human can express opinions*: human_quality_good_own_opinion
- *Human answers better than AI answers*: preferred_real
