# Supplementary material for: LLM-impersonated debate contributions are more authentic, relevant and coherent than their original: A representative study using BBC1’s Question Time
Source: PLoS One. 2026 Jul 1;21(7):e0347757. doi: 10.1371/journal.pone.0347757 (PMC13322533; doi:10.1371/journal.pone.0347757)
Supplement: S2 Appendix — (PDF) [file pone.0347757.s002.pdf]

S2 Appendix: Additional details for the results

Tables 1, 2, 3, 4, 5 and Figures 1, 2, 3, 4, 5, 6, 7 report additional details from the results of our survey.

	**Real**				**Generated**			
**Criterion**	*M*	*SD*	*MED*	*IQR*	*M*	*SD*	*MED*	*IQR*
Authenticity	0.47	0.79	0.50	1.00	0.95	0.67	1.00	1.00
Relevance	0.22	0.90	0.50	1.50	1.18	0.63	1.50	0.50
Coherence	0.15	0.90	0.00	1.50	1.13	0.65	1.00	0.50

Table 1. Additional results for Track1. The result show the parametric statistical markers mean *M*, standard deviations *SD* and the non-parametric markers median *MED* and inter-quartile range *IQR*.

**Criterion**	*M*	*SD*	*MED*	*IQR*
Authenticity	0.20	0.91	0.00	1.50
Relevance	0.68	0.80	0.50	1.50
Coherence	0.80	0.77	1.00	1.00
Content	-0.25	0.88	0.00	1.50

Table 2. Additional results for Track2. Zero indicates no difference between real and generated responses. Negative values judge real responses as better, positive values judge generated responses as better. The result show the parametric statistical markers mean *M*, standard deviations *SD* and the non-parametric markers median *MED* and inter-quartile range *IQR*.

	**Real**				**Generated**				Random			
**Criterion**	*M*	*SD*	*MED*	*IQR*	*M*	*SD*	*MED*	*IQR*	*M*	*SD*	*MED*	*IQR*
Criterion	M	SD	MED	IQR	M	SD	MED	IQR	M	SD	MED	IQR
Authenticity	0.46	0.69	0.50	1.00	0.65	0.68	0.75	1.00	0.09	0.86	0.00	1.25
Confidence	0.12	0.73	0.00	1.00	0.21	0.74	0.50	1.00	0.17	0.81	0.00	1.25

Table 3. Additional results for Track3. The result show the parametric statistical markers mean *M*, standard deviations *SD* and the non-parametric markers median *MED* and inter-quartile range *IQR*.

Criterion	M	SD	MED	IQR
I am familiar with chatbots and AI.	1.01	0.79	1.00	0.00
Chatbots and AI can provide valuable contributions to public debates.	-0.58	1.05	-1.00	1.00
I support the use of chatbots and AI in public debates.	1.45	0.69	2.00	1.00
If chatbots and AI are used, this has to be made explicit.	1.67	0.64	2.00	1.00
If chatbots and AI are used in public debates, we need to know what data the system was developed on.	1.30	0.77	1.00	1.00
Chatbots and AI should be regulated and only be employed in specific circumstances.	-0.13	1.00	0.00	2.00

Table 4. Additional results for the exit poll before revealing the use of AI. The result show the parametric statistical markers mean M, standard deviations SD and the non-parametric markers median MED and inter-quartile range IQR.

Criterion	M	SD	MED	IQR
I am familiar with chatbots and AI.	0.95	0.83	1.00	0.00
Chatbots and AI can provide valuable contributions to public debates.	-0.55	1.07	-1.00	1.00
I support the use of chatbots and AI in public debates.	1.47	0.69	2.00	1.00
If chatbots and AI are used, this has to be made explicit.	1.67	0.64	2.00	1.00
If chatbots and AI are used in public debates, we need to know what data the system was developed on.	1.31	0.77	1.00	1.00
Chatbots and AI should be regulated and only be employed in specific circumstances.	-0.06	1.03	0.00	2.00

Table 5. Additional results for the exit poll after revealing the use of AI. The result show the parametric statistical markers mean M, standard deviations SD and the non-parametric markers median MED and inter-quartile range IQR.

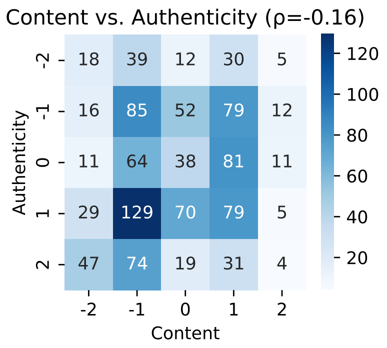

Figure 1. Judgments on the relationship between the authenticity and content when the actual response and the GPT-generated response were shown side by side. The heatmap visualises the counts of the different rating combinations. The reported statistical marker is the correlation measured with Spearman's ρ.

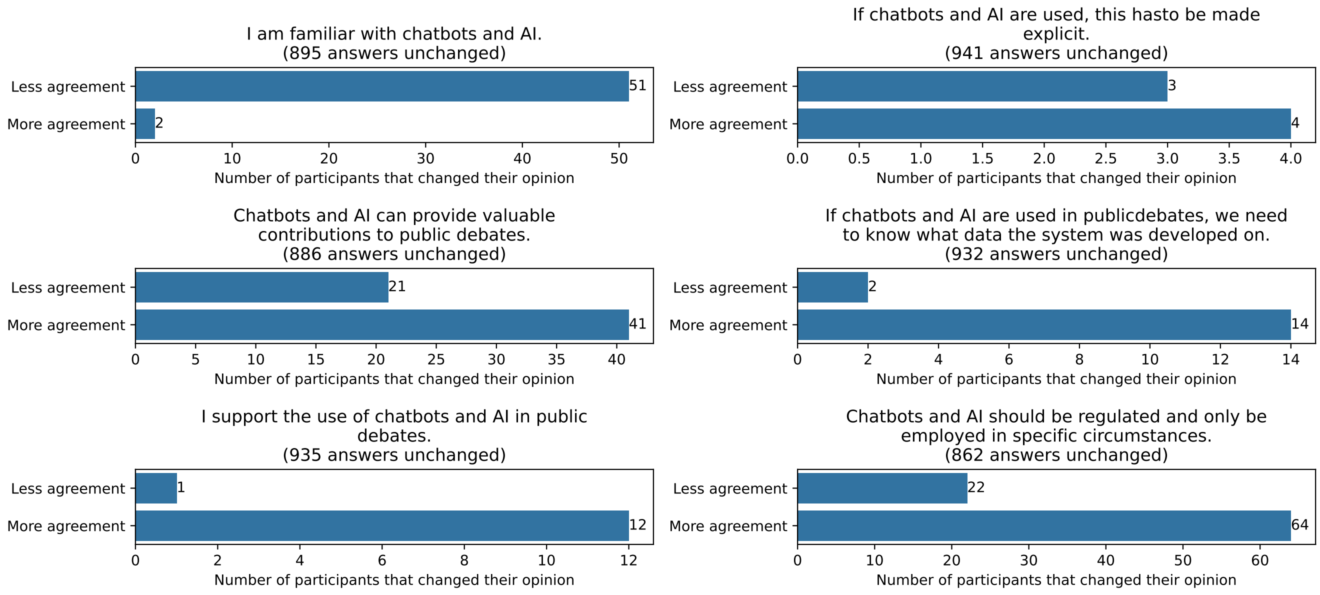

Figure 2. Changes in the participants' opinions after it was revealed which responses were AI-generated. The bar charts depict the counts of changes per question.

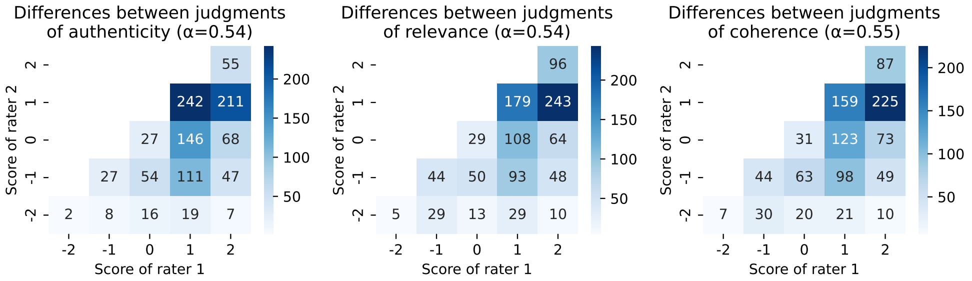

Figure 3. Inter-rater reliability when only a debate question, the name of the speaker, and either the GPT-generated response or the response of the actual speaker were shown. The heatmap visualises the counts of the different rating combinations. The reported statistical marker is Cronbach's α.

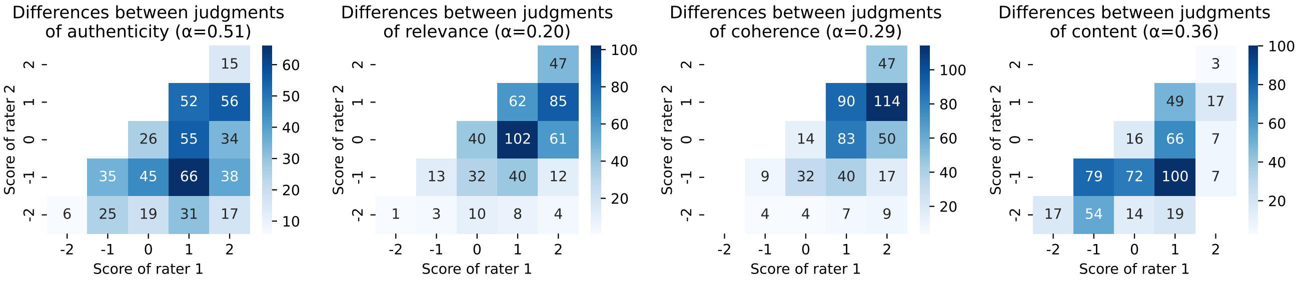

Figure 4. Inter-rater reliability when a debate question, the name of the speaker, and both the actual and GPT-generated response were shown side-by-side. The heatmap visualizes the counts of the different rating combinations. The reported statistical marker is Cronbach's α.

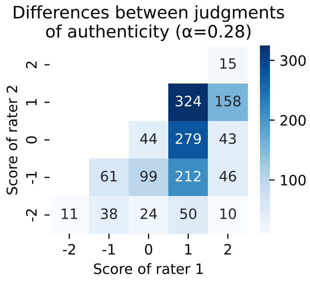

Figure 5. Inter-rater reliability when a debate question with either the response and biography from the actual speaker, the GPT-generated response and the biography of the actual speaker, or the response from the actual speaker but the biography of a random public person was shown. The heatmap visualises the counts of the different rating combinations. The reported statistical marker is Cronbach's α.

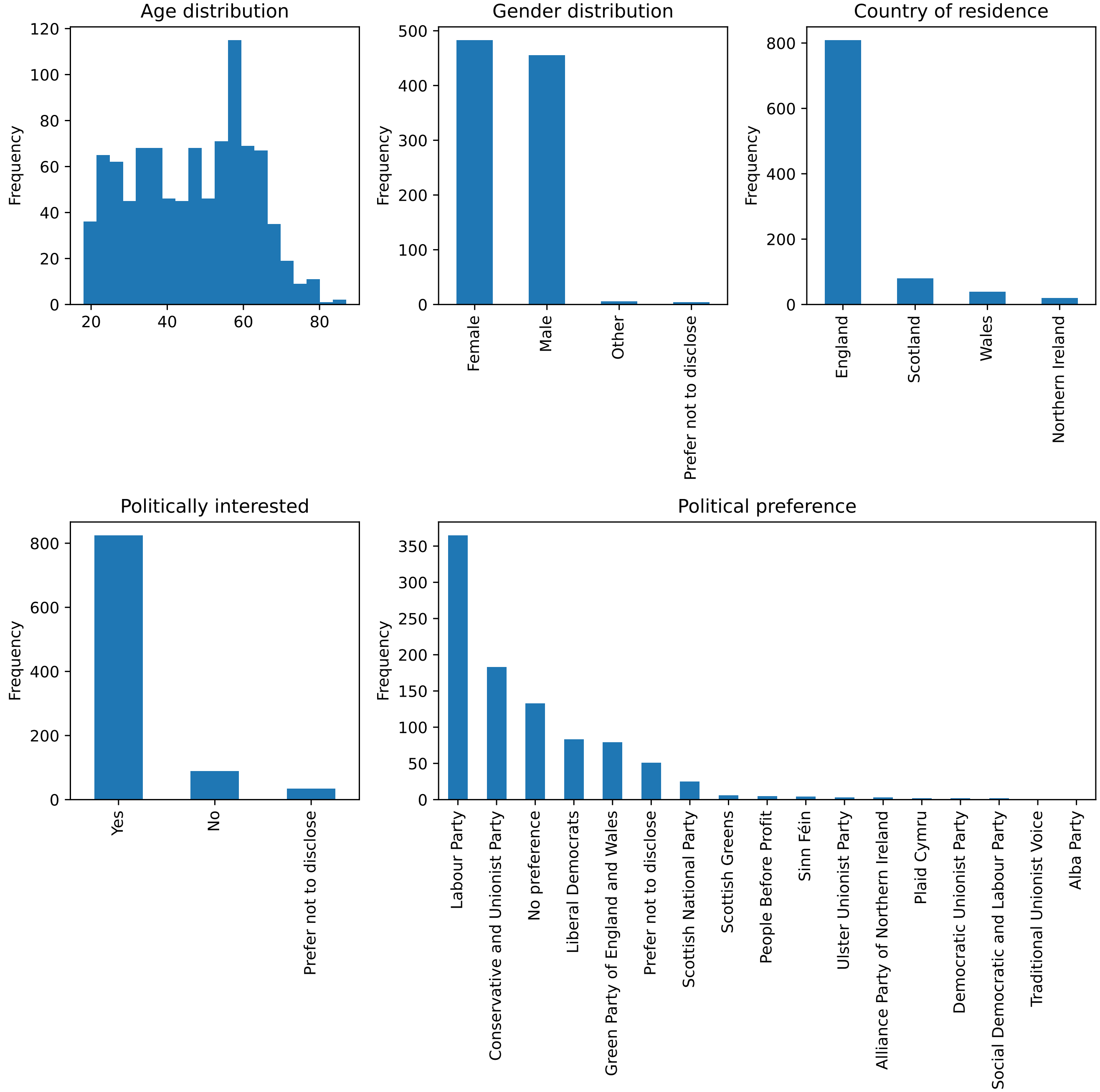

Figure 6. Demographics data of our survey participants. The histogram for the age shows the distribution of the different age categories. The bar charts for the other aspects show the counts per category.

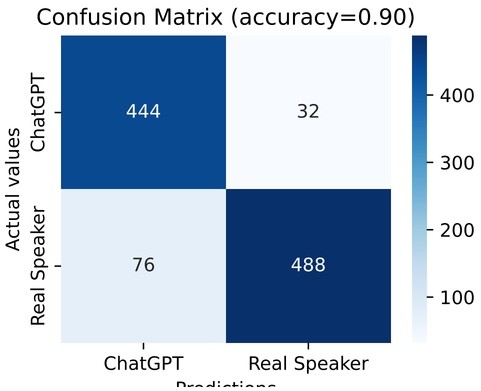

Figure 7. Confusion matrix of the automated detection of the impersonated debate responses. The accuracy is the percentage of correct results, the cells depict the counts of the respective combinations.
