# Supplementary material for: LLM-impersonated debate contributions are more authentic, relevant and coherent than their original: A representative study using BBC1’s Question Time
Source: PLoS One. 2026 Jul 1;21(7):e0347757. doi: 10.1371/journal.pone.0347757 (PMC13322533; doi:10.1371/journal.pone.0347757)
Supplement: S3 Appendix — (PDF) [file pone.0347757.s003.pdf]

S3 Appendix: Additional analysis of alternative explanations for results

In Track3, we randomly assign speakers to questions to test if authenticity is judged independent of the speaker. A possible biasing factor would be if the random assignment would lead to different frequencies of speaker assignments. Figure 1 provides additional data for this. As the plot shows, the distribution of the randomly assigned speakers is similar to the real speakers, except that it is overall a bit smoother. This indicates that there is no bias introduced by this.

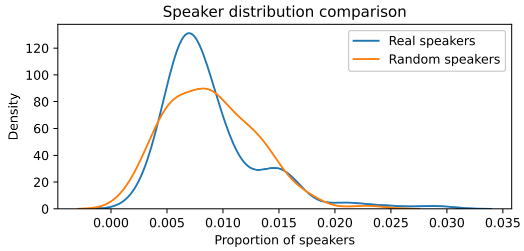

Figure 1. Frequency that speakers appear as actual speakers and as randomly assigned speakers.

In principle, judgments could be influenced by answer length. To control for this, we analyse the distribution of answer length and its correlation with the human judgments in Track1. Figure 2 shows the word count of the actual and generated answers and the statistical comparison between both. The data shows that the length is statistically significantly different with a medium effect size. However, we note that the absolute difference in length is fairly small, i.e., the real answers are on average only nine 9 words shorter. Together with the weak correlations between the word count and the human judgments (see Table 1), we can rule the answer length as alternative explanation for our results.

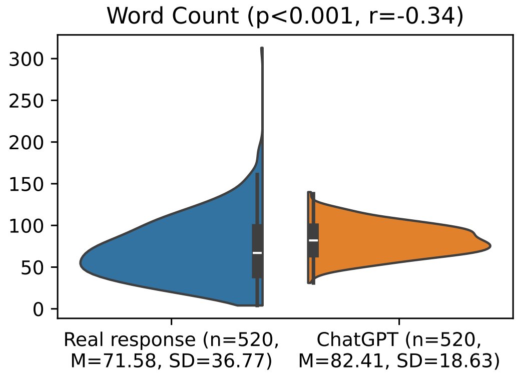

Figure 2. Word counts of generated and actual answers.
Violins show a kernel density estimation of the probability distribution, the miniature box-plots depict the median, upper and lower quartiles, and the whiskers the largest/smallest value observed within 1.5 times the interquartile range of the upper/lower quartile. The statistical markers reported are the p-value of two-sided Wilcoxon signed rank tests, the effect size *r*, the sample sizes *n*, mean values *M* and standard deviations *SD*.

Table 1. Spearman's ρ correlation between the word count and the judgments for authenticity, relevance, and coherence in Track1.

	Real response ρ	GPT-generated response ρ
Authenticity	0.13	0.01
Relevance	0.14	0.08
Coherence	0.09	0.04

The actual debate contributions are taken from verbatim transcripts of the QT30 debates. The transcription was done in real time by highly experienced professional stenographers, all British native speakers. As is the case with any live transcription,

minor flaws in the produced text are possible. In order to quantify these, we use LanguageTool[1] to automatically check both actual and generated data for British typography and grammar. Our data shows that there are significantly more spelling mistakes in the real responses than in the generated data with a large effect size (see Figure 3). However, the absolute number of mistakes is still low, even in the real responses, with a mean of 2.4 errors per real response and 0.26 per generated debate contribution.

The majority of the errors in the real responses are punctuation- and typography-related, making up about 60% of all errors (see Table 2). The punctuation issues are caused predominantly by a missing comma between the clauses of a compound sentence, while the typography issue reflects missing whitespace between the sentence-final full stop and the beginning of the next sentence. Neither of those errors affects the readability of the texts.

LanguageTool also flags a number of minor stylistic issues (about 11%) due to, for instance, repetitions, e.g., several sentences in the actual responses begin with the same word, or lexical redundancy – however, those are patterns which reflect human speech and are not grammatical errors. The actual typos and grammar mistakes make up only about 21% of the errors flagged by LanguageTool. In the generated contributions, the majority of errors (52%) are caused by the American spelling (categorised as TYPOS by LanguageTool). Lexical redundancy, punctuation, and grammar categories make up 25%, 7%, and 6% of the errors, respectively.

A correlation analysis between the spelling errors and the human judgments shows only weak correlations (see Table 3). Overall, we can rule out that the spelling errors in the texts are an alternative explanation for our results.

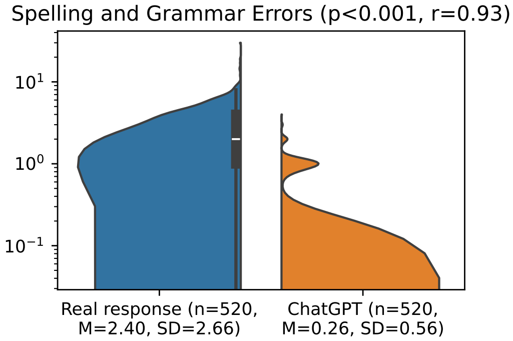

Figure 3. Spelling errors of generated and actual answers.
Violins show a kernel density estimation of the probability distribution, the miniature box-plots depict the median, upper and lower quartile, and the whiskers the largest/smallest value observed within 1.5 times the interquartile range of the upper/lower quartile. The statistical markers reported are the p-value of two-sided Wilcoxon signed rank tests, the effect size *r*, the sample sizes *n*, mean values *M* and standard deviations *SD*.

In 10% of the actual responses, we manually checked if actual responses contained references to other panellists. We found one instance (Pair ID: 39) where the speaker refers to a previous comment by another panellist ('Professor Neil Ferguson has said it'). However, given that Neil Ferguson is a prominent voice in the UK on pandemic issues, this reference does not have an impact on the meaning of the response, given that it could have been interpreted as citing other public comments by Neil Ferguson.

Table 2. Types of spelling errors detected in the responses by LanguageTool[1].

	Real response Error Count	GPT-generated response Error Count
PUNCTUATION	388	8
TYPOGRAPHY	353	0
TYPOS	170	70
GRAMMAR	91	10
REPETITIONS_STYLE	78	0
MISC	57	5
REDUNDANCY	36	32
CASING	29	1
STYLE	22	1
PROPER_NOUNS	9	5
CONFUSED_WORDS	7	1
COMPOUNDING	3	0
NONSTANDARD_PHRASES	3	0
COLLOCATIONS	2	1
MULTITOKEN_SPELLING	2	0
AMERICAN_ENGLISH	0	2

Table 3. Spearman's ρ correlation between the spelling errors and the judgments for authenticity, relevance, and coherence in Track1.

	Real response ρ	GPT-generated response ρ
Authenticity	-0.04	-0.02
Relevance	0.01	-0.08
Coherence	-0.05	-0.05

References

1. LanguageTool: Free AI Grammar Checker. https://languagetool.org/ (2025).
