# Supplementary material for: LLM-impersonated debate contributions are more authentic, relevant and coherent than their original: A representative study using BBC1’s Question Time
Source: PLoS One. 2026 Jul 1;21(7):e0347757. doi: 10.1371/journal.pone.0347757 (PMC13322533; doi:10.1371/journal.pone.0347757)
Supplement: Attachment — Submitted filename: PLOS-One-QT-GPT-Response.pdf [file pone.0347757.s004.pdf]

Response to Reviews

We thank the two reviewers, as well as the editor, for their feedback. Based on the comments, we prepared a revision of our manuscript for your consideration. To aid the reviewers, all changes are marked in blue within the updated manuscript.

Editor

Comment E.1: Reviewer 1 comments the design and analysis but raises concerns about the contextual asymmetry between the original human responses and the LLM-generated ones. Since the human content was transcribed from a live television programme ("Question Time"), it may include disfluencies, references, or transcription artifacts that affect perceptions differently than the directly generated LLM text. The reviewer suggests adding a post-hoc robustness check or qualitative analysis to demonstrate that these contextual or transcription-related differences do not drive the main effects.

Response E.1: We address this issue by conducting additional checks such as automated spell checking and a manual analysis of the responses to rule out such effects. The additional results are reported in the new supplemental material S3.

For more details, we refer to Response R1.1.

Comment E.2: Relatedly, Reviewer 1 encourages you to expand the discussion of content differences between human and impersonated responses. Specifically, the paper would benefit from showing how these differ substantively; for example, by analyzing whether impersonations diverge in stance or argumentation, rather than merely in surface-level content. This would clarify whether the framing of "misrepresentation" is warranted.

Response E.2: Based on these understandable concerns, we conduct additional manual analysis, in which we compare the stance conveyed by the actual and the generated response. While there are indeed cases in which the differences are rather in how a stance is conveyed, we also find a non-negligible number (26%) of the samples that we checked, where the differences in content were indeed differences in the stance that was conveyed.

For more details, we refer to Response 1.2.

Comment E.3: Finally, the reviewer notes that the reliability of human ratings is modest and recommends greater transparency about inter-rater variability, or, if feasible, additional averaging or collection of ratings to reduce subjective noise.

Response E.3: While we acknowledge this concern, we want to highlight that the differences in the judgments are neither random (i.e., completely unreliable) nor polar (i.e., opposite judgments), but rather on neighboring items on the Likert scale. We discuss these concerns in detail in the "Human judgment is reliable" section, which dedicates a whole

paragraph to this issue. While we acknowledge that additional data collection could possibly help, we do not believe that there will be substantial changes, given our already very large sample size of 520 question/response pairs.

For more details, we refer to Response 1.3.

Comment E.4: Minor notes include adding a brief explanation that Question Time is a television programme, clarifying “random speaker” in the methods, and checking for potential answer length differences that could influence results.

Response E.4: We now address all these concerns explicitly in the paper, including an additional analysis on the random speaker assignments and the influence of answer lengths.

For more details, we refer to Responses 1.4, 1.5, and 1.6.

Comment E.5: 2. Conceptual and methodological refinements (Reviewer 2)

Reviewer 2 finds the study compelling but identifies several areas where methodological clarification is needed:

Construct clarity: The meaning of authenticity should be defined more precisely, whether it captures authorship likelihood, realism, or plausibility. Reviewers recommend clarifying how this construct differs from related ones (e.g., coherence or content similarity) and ensuring that participants understood it consistently.

Response E.5: We clarify our definition for authenticity as requested.

For more details, refer to Response 2.1.

Comment E.6: Experimental design: Provide more detail on the randomization and balancing of stimuli across conditions. If possible, include or reference any inter-rater checks used during the manual screening of generated responses.

Response E.6: We acknowledge that we should have been clearer regarding these issues. We almost always use the full data set and avoid sampling. The only case where we sample is the random assignment of speakers, as well as the new qualitative analysis of answers we already discussed above. We provide additional analysis for this aspect. We also failed to clarify why no inter-rater agreements are reported for our own manual analysis: all cases in which the second rater disagreed with the original judgment by the first rater were discussed and agreement was reached in all cases.

For more details, we refer to Responses 2.3 and 2.4.

Comment E.7: Measurement approach: Consider complementing subjective Likert judgments with an objective or behavioral indicator (e.g., response times or accuracy), or at least acknowledge the limitations of relying solely on self-reported data.

Response E.7: While this would yield insightful additional data, such data cannot be reliably obtained with the only survey method we used. While there is possibly an impact because of unobserved confounding effects, this is highly unlikely given our sample size. Still, we acknowledge this limitation in the “Human judgments are reliable” section.

For more details we refer to Response 2.5.

Comment E.8: Statistical analysis: The reviewer suggests using non-parametric or mixed-effects models better suited to ordinal and nested data, reporting medians and interquartile ranges, and revising effect size measures accordingly. You might also re-evaluate inter-rater reliability using intraclass correlation coefficients.

Response E.8: We carefully considered all suggestions by the reviewer and partially updated our methods. We revised the effect sizes to be non-parametric (almost no changes, one increase in in effect strength). All non-parametric markers are now reported next to the parametric mean and standard deviation in the appendix and we explain why we kept the mean and standard deviation in the main body of the paper. We did not switch to a different statistical model (i.e., no variant of a linear model), because we directly want to observe how the variables change and not how the combination of variables explains a change in dependent variable.

For more details, we refer to Response 2.6.

Comment E.9: Interpretation: Finally, temper the strength of the claims about deception or indistinguishability between AI and human responses. Reviewer 2 encourages reframing your findings as perceptual rather than deceptive effects, potentially driven by linguistic fluency or stylistic uniformity.

Response E.9: While our additional analysis further supports our results, we acknowledge that some wordings were too strong in the initial submission as our results are limited to the perception of our participants. We consequently edited the wording in key locations of the paper such as the abstract and the results section.

For more details, we refer to Response 2.7.

Journal Requirements:

Comment J.1: When submitting your revision, we need you to address these additional requirements.

Please ensure that your manuscript meets PLOS ONE's style requirements, including those for file naming. The PLOS ONE style templates can be found at https://journals.plos.org/plosone/s/file?id=wjVg/PLOSOne_formatting_sample_main_body.pdf and https://journals.plos.org/plosone/s/file?id=ba62/PLOSOne_formatting_sample_title_authors_affiliations.pdf

Response J.1: We updated all file names and types to match the requirements.

Comment J.2: Please update your submission to use the PLOS LaTeX template. The template and more information on our requirements for LaTeX submissions can be found at http://journals.plos.org/plosone/s/latex.

Response J.2: We updated our submission to use the latest version of the LaTeX style file (Version 3.7 from Aug 2025)

Comment J.3: We note that the grant information you provided in the 'Funding Information' and 'Financial Disclosure' sections do not match.

When you resubmit, please ensure that you provide the correct grant numbers for the awards you received for your study in the 'Funding Information' section.

Response J.3: Thank you for reporting this. We now clarify the matter in the cover letter.

Comment J.4: Thank you for stating the following financial disclosure:
A.H. work was partially funded by the VolkswagenStiftung under grant Az. 98544 'Deliberation Laboratory'
URL: https://www.volkswagenstiftung.de/
The funders had no role in the study whatsoever.

Please state what role the funders took in the study. If the funders had no role, please state: "The funders had no role in study design, data collection and analysis, decision to publish, or preparation of the manuscript."
If this statement is not correct you must amend it as needed.
Please include this amended Role of Funder statement in your cover letter; we will change the online submission form on your behalf.

Response J.4: The funders had no role in the study and we clarify this in the cover letter.

Comment J.5: Thank you for stating the following in the Acknowledgments Section of your manuscript:
The work reported on in this paper was partially funded by the VolkswagenStiftung under grant Az. 98544 'Deliberation Laboratory'

We note that you have provided funding information that is not currently declared in your Funding Statement. However, funding information should not appear in the Acknowledgments section or other areas of your manuscript. We will only publish funding information present in the Funding Statement section of the online submission form. Please remove any funding-related text from the manuscript and let us know how you would like to update your Funding Statement. Currently, your Funding Statement reads as follows:
A.H. work was partially funded by the VolkswagenStiftung under grant Az. 98544 'Deliberation Laboratory'
URL: https://www.volkswagenstiftung.de/
The funders had no role in the study whatsoever.

Please include your amended statements within your cover letter; we will change the online submission form on your behalf.

Response J.5: Thank you for this clarification. We provide the amended statements in our cover letter and drop the acknowledgements section from the manuscript.

Comment J.6: Thank you for uploading your study's underlying data set. Unfortunately, the repository you have noted in your Data Availability statement does not qualify as an acceptable data repository according to PLOS's standards.

At this time, please upload the minimal data set necessary to replicate your study's findings to a stable, public repository (such as figshare or Dryad) and provide us with the relevant URLs, DOIs, or accession numbers that may be used to access these data. For a list of recommended repositories and additional information on PLOS standards for data deposition, please see https://journals.plos.org/plosone/s/recommended-repositories.

Response J.6: We noticed that the DOI-reference to our long-term archive at Zenodo was missing the last character. This is now fixed and the new Data Availability statement is:
"All materials are available online in the form of a replication package that contains the data: https://doi.org/10.5281/zenodo.12698364"

Comment J.7: Please include your full ethics statement in the 'Methods' section of your manuscript file. In your statement, please include the full name of the IRB or ethics committee who approved or waived your study, as well as whether or not you obtained informed written or verbal consent. If consent was waived for your study, please include this information in your statement as well.

Response J.7: We now have an additional subsection at the end of the Methods section with the full ethics statement. We extend the ethics statement to clarify that the obtained consent was written.

Comment J.8: If the reviewer comments include a recommendation to cite specific previously published works, please review and evaluate these publications to determine

whether they are relevant and should be cited. There is no requirement to cite these works unless the editor has indicated otherwise.

Response J.8: Thank you for this comment. There were no recommendations for additional references in the reviews.

Reviewer 1

Comment R1.1: QT is a television programme, where panellists give live answers to audience questions. This means that the real human responses that form the basis of this paper are transcribed from speech that occurred in a specific context. The LLM, on the other hand, generated text directly in a decontextualised setting, as provided by the system prompt (lines 84ff).
The authors acknowledge this discrepancy (e.g. line 443) but do not meaningfully engage with it. Fundamentally, human responses are not being evaluated in the setting and modality for which they were intended.
The paper would be stronger if it demonstrated that this discrepancy does not affect results. For example, human responses may include markers (such as references to other panellists names, disfluencies) that are perceived as inauthentic, incoherent, etc. when transcribed and decontextualised, but not when spoken on the programme. There may also be transcription errors, which LLMs would not have. This seems in scope for post-hoc analysis.

Response R1.1: This is a good point and would be a viable alternative explanation for our results. We add additional analysis regarding this (and other) alternative hypothesis to the supplemental material in Appendix S3. We decided to exclude this from the main body of the paper in order to keep the presentation crisp and only add a reference to this new supplemental material to the end of the Section “Statistical analysis”. In summary, errors have no correlation with judgments. While transcriptions have more errors (2.4 on average vs. 0.26 for generated responses), most errors are minor, e.g., missing commas between clauses of compound sentences. In 10% of the original responses, we manually checked if actual responses contain references to other panelists and we do not find such cases. We add this information in Section “Original Debate Content.”

Comment R1.2: The paper finds that original content is different from impersonated content and concludes from this that AI may be used to generate targeted misinformation about the speaker’s point of view (lines 339ff). However, there is no analysis into *how* content differs in human vs LLM-impersonated content, and whether this difference does in fact correspond to “misrepresentation”. For this, it would be necessary to show that the LLM-impersonated answers differ from the human answers in their issue-specific stance.
It seems very plausible that LLMs follow different lines of argumentation but ultimately make similar points, which may (hypothetically) even be endorsed by the original speaker. Asking only about “difference in content”, which may be explained in many ways, cannot capture this. Consequently, the framing of the paper as relating to misinformation and deception seems only partly warranted.

Response R1.2: Thank you for this comment. We acknowledge that our definition of the term "content" as "difference in overall meaning" is too broad to directly justify these statements. To mitigate this, we include additional qualitative analysis on the share of data that was judged as different in content, indicated by a negative mean value of the "content" judgments by the study participants. The goal of this qualitative analysis is to establish whether the differences in content are indeed about misrepresentation. For this, we focus on the stance, i.e., if the response arrives at the same conclusion, argues for the same points or takes the same stance. This focus excludes the use of rhetorical devices as other means to convey a point, which may be used to make misrepresentations authentic. Additionally, we check if the responses actually address the question, as dodging the actual question could also be an important reason for differences in content. Overall, we analyze 50 response-pairs that were judged as different in content. We extend the section "Original content is different from impersonated content" with this analysis, showing that while 42% of differences are only due to how a position is presented, there are 32% of cases in which one side dodges the question (mostly the actual speakers) and, crucially, 26% where there is indeed a difference in stance that is conveyed, highlighting the potential for misinformation.

Comment R1.3: The paper claims that human judgments are reliable but does not provide strong evidence for this. Agreement is modest at best (lines 359 following), and the supplementary variables show low agreement on several variables. Clearly, there is substantial noise in the data from subjective rating differences, which could be addressed by collecting and averaging across more ratings. If not, it would still be useful to be more open and specific about the (lack of) human agreement in the main body of the paper.

Response R1.3: While we acknowledge that the absolute agreement (i.e., exactly the same value) is only modest, we provide detailed data that the disagreements are not random (i.e., judgments would be completely unreliable) or polar (i.e., raters often judging the opposite of each other), but rather that disagreements are on neighboring items on the scale.

Moreover, we respectfully disagree that we are not discussing this sufficiently in the main body of the paper. Indeed, the section "Human judgment is reliable" has a whole paragraph dedicated to describing the differences in human judgments, first highlighting the modest overall agreement, followed by describing the qualitative aspects of the disagreement, concluding with why we believe that the data supports that the data is, indeed, reliable.

Additional data collection could - possibly - mitigate this somewhat, but given our already very large sample of data with two judgments for each of 520 question/response pairs in three tracks, we do not believe that there will be substantial changes. Finally, we want to highlight the very high costs associated with collecting this amount of data from a representative cross-section of society, making a large increase in sample size (e.g., three or four judgments per data point) infeasible.

Comment R1.4: I found no mention of answer length in the paper. It seems very plausible that there is a difference in length between human and LLM answers, which may in turn affect at least some of the analyses. Some normalisation would likely be necessary?

Response R1.4: We add the missing data regarding answer length to the supplemental material (S3). While answer lengths are significantly different, the average difference is only nine words. Moreover, we only find weak correlations between answer lengths and human judgments, which indicates that this is not an alternative explanation.
The linguistic data for modals, discourse markers, epistemic markers, nominalizations, and lexical markers are already normalized by the number of sentences within a response to avoid length-based effects. We now add a clarifying statement to the paper where these variables are introduced (Section 'Stylistic comparisons').

Comment R1.5: It may help the (non-UK) reader to note in the Data section that Question Time is a TV programme. The only mention of TV / television right now, I believe, is in the intro.

Response R1.5: We have extended the description in the 'Original debate content' section after line 58 to emphasize this fact.

Comment R1.6: On first reading, I was a bit confused by lines 117-119: "random speaker" may need a brief explanation. Of course, this becomes clear later.

Response R1.6: Thank you for this point. We have reformulated this information in lines 119 and 120.

Reviewer 2

Comment R2.1: Experimental Design
The overall three-track design is conceptually sound allowing the author to assess perceptions under different conditions. Nonetheless, the construct of authenticity is insufficiently defined. It remains unclear whether participants interpreted it as authorship likelihood, realism, or plausibility, which may conflate distinct psychological dimensions.
Suggestion: Clarify the exact wording and intended meaning of each measure, particularly "authenticity."

Response R2.1: Thank you for this comment. For authenticity, the Cambridge dictionary lists as the description "the quality of being real or true", which we use to slightly adjust the wording of this dimension in the paper, from 'actual utterance' to 'actual and real utterance'. For the dimensions 'Coherence' and 'Relevance', we do not see how these require further clarification.

Comment R2.2: Moreover, each participant evaluated only a few items, which limits the reliability of individual responses.

Response R2.2: We respectfully disagree with this concern. That each participant only judges a relatively small set of items should only have a strong effect for tasks with a

learning curve. We see no reason for such a learning curve to exist for judgments of authenticity, coherence, relevance, similarity of content, or familiarity with speakers.

Comment R2.3: Besides, more details about the randomization of questions and speakers is required to ensure data is balanced.
Suggestion: Provide evidence that the randomization of stimuli was balanced across conditions.

Response R2.3: For each track and rating, we used the full data set that was available, i.e., all 520 question/response pairs. The only random element that may affect our results is the selection of random speakers within Track 3. Here, we use simple random sampling from all speakers. We acknowledge that we have not reported any checks on whether this sampling may have led to a biased distribution. To test for this, we compare the distribution of real speakers with the distribution of randomly assigned speakers.

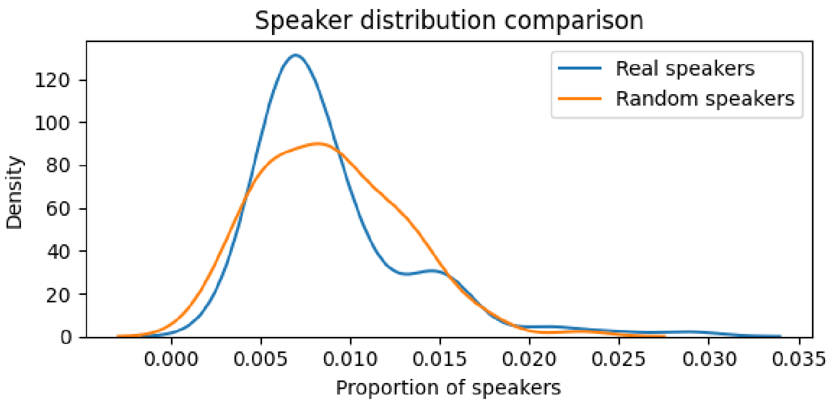

As the plot shows, the distribution of the randomly assigned speakers is similar to the real speakers, except that it is overall a bit smoother. We extended the supplemental material with this information (S3).

Comment R2.4: The manual inspection of generated responses lacks formal coding criteria or inter-rater checks, reducing transparency and reproducibility.
Suggestion: Include or reference inter-rater checks for the manual screening of generated responses.

Response R2.4: Thank you for this comment. The coding of the generated responses is not done independently by multiple annotators, but rather together by two authors in a manner that ensures adjudication. While one author assigns all codes independently first, a second author fully checks all results and comments on all disagreements. Instead of using both, possibly diverging annotations, the authors then discuss all disagreements to come to a commonly agreed upon labeling. Consequently, measuring the inter-rater agreement is not reasonable here. We add a clarifying statement to the manuscript: "*We do not report inter-rater agreements for this data, because all data was fully checked by two authors and*

all disagreements were discussed and resolved, which results in final coding results to which both annotators perfectly agree."

Comment R2.5: Measures and Data Collection
The study relies entirely on self-reported Likert-scale judgments. This approach is valid for perceptual studies, but complementary behavioral or comprehension-based measures (e.g., response time, detection accuracy) would have strengthened construct validity. Some measures—especially "content similarity"—may overlap conceptually with "authenticity," potentially introducing redundancy.

Suggestions for improvement:
Consider clarifying the independence of constructs and, if possible, provide an additional objective indicator of perceptual discrimination between real and impersonated responses. Provide information about possible moderator factors.

Response R2.5: While we acknowledge the utility of such measures, a reliable measurement is not possible in the online setup we use. There are many confounding factors affecting any measurements of timing, e.g., ranging from planned interruptions (e.g., participants doing multiple studies on Prolific that schedule breaks in fixed intervals, which might be during the completion of a survey), unplanned interruptions (e.g., due to technical issues or family members coming into the room) or similar. There are even cases where we had to resolve technical problems from participants due to their own non-standard setups. While we recorded timing data nevertheless, the quality of this data is of such questionable nature, that we cannot reasonably use this as an additional variable for our analysis.

While we acknowledge that for instance the capabilities of the participants and their ability to comprehend texts could be a moderating factor, this should only have an effect if it was unevenly distributed across different data within the tracks, e.g., in Track1, participants with low reading comprehension skills could have had more real responses assigned. However, this is highly unlikely in our setting due to the very large sample size of 520 question/answer pairs. Consequently, while we cannot rule out such a moderating factor with absolute certainty, the likelihood of such an unbalanced sampling of participants with a sample size of 520 questions is extremely low. Further, each participant rated, on average, half of the questions from both real and actual speakers, further reducing the likelihood of such an effect.

Nevertheless, even though we have good reasons to believe that our results are sound, we now acknowledge this limitation in Section "Human judgments are reliable".

Comment R2.6: Statistical Analysis
The paper presents a detailed description of the statistical workflow and justifies the use of non-parametric tests. However, several inconsistencies remain. The analysis averages ordinal Likert data and reports means, standard deviations, and Cohen's d, assuming interval properties. This is not strictly correct for ranked data and can inflate effect sizes. Inter-rater reliability (reported Cronbach's α) indicates moderate agreement at best, suggesting considerable noise in the judgments. The treatment of each outcome variable

(authenticity, coherence, relevance, content) as independent is also problematic, as they are conceptually and statistically correlated. Finally, while some corrections are applied, the overall analytical structure would benefit from mixed-effects or ordinal regression models that account for the nested and repeated nature of the data.
Suggestions for improvement:
Report medians and interquartile ranges alongside or instead of means. Use effect size measures suited for ordinal data (e.g., rank-biserial correlation or other relevant measure.) Reassess reliability using intraclass correlation or a mixed-model framework. Consider a multivariate or mixed-effects approach to handle correlated measures and participant/item dependencies.

Response R2.6: We note that while we use variables that are based on Likert scales, our statistical analysis and reporting is not directly on the individual judgments, but rather on the mean of the two judgments per data point, based on mapping the Likert scales to points [-2, -1, 0, 1, 2]. However, we acknowledge that this still results in a similarly discrete scale that just adds .5-values. Consequently, we replace Cohen's d with the rank-biserial correlation as suggested, computed from the p-values of the Wilcoxon tests (Track1, Track2), resp. Mann-Whitney-U tests (for Track3). We use the common approach of $r<0.3$ for a small effect, $0.3<=r<0.5$ for medium effect, and $r>0.5$ for a large effect.

There are no major changes to our results based on this change. In one case the strength of the effect changes from medium to large, i.e. for the authenticity in Track1.

We decided to keep reporting the mean and standard deviation in the main body of the paper, due to the reason previously provided: The distribution of the data when mapped to the points looks mostly normal and the mean and standard deviation are easy to interpret. Moreover, the reporting of the mean value has the advantage that we see if the population if the central tendency is shifted to the negative or positive from zero, as, e.g., no difference between 0.0 and 0.5 is visible when using the medians. Nevertheless, we agree that we should report the non-parametric in comparison to the parametric markers. This is already the case in the visualizations in the main body that contain miniature boxplots that depict the median and upper/lower quartiles. Additionally, we include new tables (Tables 1-5 in supplemental material S2) that contain the mean values, standard deviations, medians, and inter-quartile ranges for all results.

Finally, we acknowledge that a multivariate analysis, e.g., for Track1 with a linear model that uses whether the data is generated or not as dependent variable, logit as link function, and authenticity, relevance, and coherence as independent variables. We agree that such a model has the advantage that the correlations between the independent variables are considered when computing the coefficients. However, the interpretation of the coefficients would not yield what we are interested in, which is to directly measure how the central tendency of the independent variables is shifted between the two populations of real and generated responses, which is exactly what the Wilcoxon tests determine the significance for. In other words, we do not want to measure how authenticity changes when we control for relevance and coherence, but rather how it changes absolutely. The same applies to the other variables. Consequently, we do not switch the statistical framework to a linear model or similar.

Comment R2.7: Interpretation of Results
The finding that impersonated responses are perceived as more authentic, coherent, and relevant than the originals is statistically supported but likely overstated. Given the modest reliability and limited item sampling, the conclusion that participants “cannot discern” AI-generated content should be presented more cautiously. It is plausible that judgments reflect linguistic fluency rather than genuine attribution of authorship.
Suggestions for improvement:
Moderate the interpretive claims and emphasize the perceptual, rather than deceptive, nature of the results. Discuss alternative explanations such as stylistic consistency or linguistic polish in LLM outputs.

Response 2.7: We tone down this claim across the paper, indeed including the perceptive element of our study, see the amended sentence in the abstract and the revised heading in the results section (‘Impersonated responses are *perceived as* more authentic, coherent and relevant’). Regarding alternative interpretations, we refer to the additional analysis provided based on hints from Reviewer 1 (see R1.1 and R1.2) which are now discussed in supplemental material S3.
