# Supplementary material for: LLM-impersonated debate contributions are more authentic, relevant and coherent than their original: A representative study using BBC1’s Question Time
Source: PLoS One. 2026 Jul 1;21(7):e0347757. doi: 10.1371/journal.pone.0347757 (PMC13322533; doi:10.1371/journal.pone.0347757)
Supplement: Peer review history

# Peer review: Decision Letter 0

**DOI:** 10.1371/journal.pone.0347757.r001

**Authors:** Carlos Carrasco-Farré

19 Oct 2025

PONE-D-25-15257

LLM-impersonated debate contributions are more authentic, relevant and coherent than their original: A representative study using BBC1’s Question Time

PLOS ONE

Dear Dr. Herbold,

Thank you for submitting your manuscript to *PLOS ONE*   . I am pleased to inform you that, following peer review, the reviewers and I found your study to be original, clearly written, and addressing a timely and important topic. Both reviewers recognized the relevance and ambition of your work and appreciated its methodological clarity and potential contribution.. I am pleased to inform you that, following peer review, the reviewers and I found your study to be original, clearly written, and addressing a timely and important topic. Both reviewers recognized the relevance and ambition of your work and appreciated its methodological clarity and potential contribution.

==============================

However, they also identified several issues that need to be addressed before the manuscript can be considered for publication. Based on their feedback, I am inviting you to **submit a major revision**   . Below, I summarize the main points raised by the reviewers and provide some guidance on how to proceed.. Below, I summarize the main points raised by the reviewers and provide some guidance on how to proceed.

<h3 data-end="1014" data-start="953">**1. Contextual and interpretive alignment (Reviewer 1)**   </h3></h3>

Reviewer 1 commends the design and analysis but raises concerns about the contextual asymmetry between the original human responses and the LLM-generated ones. Since the human content was transcribed from a live television programme (“Question Time”), it may include disfluencies, references, or transcription artifacts that affect perceptions differently than the directly generated LLM text. The reviewer suggests adding a post-hoc robustness check or qualitative analysis to demonstrate that these contextual or transcription-related differences do not drive the main effects.

Relatedly, Reviewer 1 encourages you to expand the discussion of content differences between human and impersonated responses. Specifically, the paper would benefit from showing *how*    these differ substantively; for example, by analyzing whether impersonations diverge in stance or argumentation, rather than merely in surface-level content. This would clarify whether the framing of “misrepresentation” is warranted.these differ substantively; for example, by analyzing whether impersonations diverge in stance or argumentation, rather than merely in surface-level content. This would clarify whether the framing of “misrepresentation” is warranted.

Finally, the reviewer notes that the reliability of human ratings is modest and recommends greater transparency about inter-rater variability, or, if feasible, additional averaging or collection of ratings to reduce subjective noise.

Minor notes include adding a brief explanation that *Question Time*    is a television programme, clarifying “random speaker” in the methods, and checking for potential answer length differences that could influence results.is a television programme, clarifying “random speaker” in the methods, and checking for potential answer length differences that could influence results.

<h3 data-end="2561" data-start="2496">**2. Conceptual and methodological refinements (Reviewer 2)**   </h3></h3>

Reviewer 2 finds the study compelling but identifies several areas where methodological clarification is needed:

- **Construct clarity:**    The meaning of The meaning of *authenticity*    should be defined more precisely, whether it captures authorship likelihood, realism, or plausibility. Reviewers recommend clarifying how this construct differs from related ones (e.g., coherence or content similarity) and ensuring that participants understood it consistently.should be defined more precisely, whether it captures authorship likelihood, realism, or plausibility. Reviewers recommend clarifying how this construct differs from related ones (e.g., coherence or content similarity) and ensuring that participants understood it consistently.
- **Experimental design:**    Provide more detail on the randomization and balancing of stimuli across conditions. If possible, include or reference any inter-rater checks used during the manual screening of generated responses.Provide more detail on the randomization and balancing of stimuli across conditions. If possible, include or reference any inter-rater checks used during the manual screening of generated responses.
- **Measurement approach:**    Consider complementing subjective Likert judgments with an objective or behavioral indicator (e.g., response times or accuracy), or at least acknowledge the limitations of relying solely on self-reported data.Consider complementing subjective Likert judgments with an objective or behavioral indicator (e.g., response times or accuracy), or at least acknowledge the limitations of relying solely on self-reported data.
- **Statistical analysis:**    The reviewer suggests using non-parametric or mixed-effects models better suited to ordinal and nested data, reporting medians and interquartile ranges, and revising effect size measures accordingly. You might also re-evaluate inter-rater reliability using intraclass correlation coefficients.The reviewer suggests using non-parametric or mixed-effects models better suited to ordinal and nested data, reporting medians and interquartile ranges, and revising effect size measures accordingly. You might also re-evaluate inter-rater reliability using intraclass correlation coefficients.
- **Interpretation:**    Finally, temper the strength of the claims about deception or indistinguishability between AI and human responses. Reviewer 2 encourages reframing your findings as perceptual rather than deceptive effects, potentially driven by linguistic fluency or stylistic uniformity.Finally, temper the strength of the claims about deception or indistinguishability between AI and human responses. Reviewer 2 encourages reframing your findings as perceptual rather than deceptive effects, potentially driven by linguistic fluency or stylistic uniformity.

Both reviewers found your study promising and well written. Their feedback is intended to help you enhance the rigor, clarity, and interpretive precision of your work. I encourage you to take this as a positive step toward publication; since the paper already has a strong foundation and, with the recommended revisions, could make a meaningful contribution to ongoing debates on authenticity, impersonation, and LLM-mediated communication.

==============================

Please submit your revised manuscript by Dec 03 2025 11:59PM. If you will need more time than this to complete your revisions, please reply to this message or contact the journal office at plosone@plos.org. When you're ready to submit your revision, log on to https://www.editorialmanager.com/pone/ and select the 'Submissions Needing Revision' folder to locate your manuscript file.. When you're ready to submit your revision, log on to https://www.editorialmanager.com/pone/ and select the 'Submissions Needing Revision' folder to locate your manuscript file.

Please include the following items when submitting your revised manuscript:

- A rebuttal letter that responds to each point raised by the academic editor and reviewer(s). You should upload this letter as a separate file labeled 'Response to Reviewers'.
- A marked-up copy of your manuscript that highlights changes made to the original version. You should upload this as a separate file labeled 'Revised Manuscript with Track Changes'.
- An unmarked version of your revised paper without tracked changes. You should upload this as a separate file labeled 'Manuscript'.

If you would like to make changes to your financial disclosure, please include your updated statement in your cover letter. Guidelines for resubmitting your figure files are available below the reviewer comments at the end of this letter.

If applicable, we recommend that you deposit your laboratory protocols in protocols.io to enhance the reproducibility of your results. Protocols.io assigns your protocol its own identifier (DOI) so that it can be cited independently in the future. For instructions see: https://journals.plos.org/plosone/s/submission-guidelines#loc-laboratory-protocols. Additionally, PLOS ONE offers an option for publishing peer-reviewed Lab Protocol articles, which describe protocols hosted on protocols.io. Read more information on sharing protocols at . Additionally, PLOS ONE offers an option for publishing peer-reviewed Lab Protocol articles, which describe protocols hosted on protocols.io. Read more information on sharing protocols at https://plos.org/protocols?utm_medium=editorial-email&utm_source=authorletters&utm_campaign=protocols..

We look forward to receiving your revised manuscript.

Kind regards,

Carlos Carrasco-Farré

Academic Editor

PLOS ONE

**Journal Requirements:**

1. When submitting your revision, we need you to address these additional requirements.

Please ensure that your manuscript meets PLOS ONE's style requirements, including those for file naming. The PLOS ONE style templates can be found at

https://journals.plos.org/plosone/s/file?id=wjVg/PLOSOne_formatting_sample_main_body.pdf and

https://journals.plos.org/plosone/s/file?id=ba62/PLOSOne_formatting_sample_title_authors_affiliations.pdf

2. Please update your submission to use the PLOS LaTeX template. The template and more information on our requirements for LaTeX submissions can be found at http://journals.plos.org/plosone/s/latex.

3. We note that the grant information you provided in the ‘Funding Information’ and ‘Financial Disclosure’ sections do not match.

When you resubmit, please ensure that you provide the correct grant numbers for the awards you received for your study in the ‘Funding Information’ section.

4. Thank you for stating the following financial disclosure:

A.H. work was partially funded by the VolkswagenStiftung under grant Az. 98544 ‘Deliberation Laboratory’

URL: https://www.volkswagenstiftung.de/

The funders had no role in the study whatsoever.

Please state what role the funders took in the study.  If the funders had no role, please state: "The funders had no role in study design, data collection and analysis, decision to publish, or preparation of the manuscript."

If this statement is not correct you must amend it as needed.

Please include this amended Role of Funder statement in your cover letter; we will change the online submission form on your behalf.

5. Thank you for stating the following in the Acknowledgments Section of your manuscript:

The work reported on in this paper was partially funded by the VolkswagenStiftung under grant Az. 98544 ‘Deliberation Laboratory’

We note that you have provided funding information that is not currently declared in your Funding Statement. However, funding information should not appear in the Acknowledgments section or other areas of your manuscript. We will only publish funding information present in the Funding Statement section of the online submission form.

Please remove any funding-related text from the manuscript and let us know how you would like to update your Funding Statement. Currently, your Funding Statement reads as follows:

A.H. work was partially funded by the VolkswagenStiftung under grant Az. 98544 ‘Deliberation Laboratory’

URL: https://www.volkswagenstiftung.de/

The funders had no role in the study whatsoever.

Please include your amended statements within your cover letter; we will change the online submission form on your behalf.

6. Thank you for uploading your study's underlying data set. Unfortunately, the repository you have noted in your Data Availability statement does not qualify as an acceptable data repository according to PLOS's standards.

At this time, please upload the minimal data set necessary to replicate your study's findings to a stable, public repository (such as figshare or Dryad) and provide us with the relevant URLs, DOIs, or accession numbers that may be used to access these data. For a list of recommended repositories and additional information on PLOS standards for data deposition, please see https://journals.plos.org/plosone/s/recommended-repositories.

7. Please include your full ethics statement in the ‘Methods’ section of your manuscript file. In your statement, please include the full name of the IRB or ethics committee who approved or waived your study, as well as whether or not you obtained informed written or verbal consent. If consent was waived for your study, please include this information in your statement as well.

8. If the reviewer comments include a recommendation to cite specific previously published works, please review and evaluate these publications to determine whether they are relevant and should be cited. There is no requirement to cite these works unless the editor has indicated otherwise.

[Note: HTML markup is below. Please do not edit.]

Reviewers' comments:

Reviewer's Responses to Questions

**Comments to the Author**

1. Is the manuscript technically sound, and do the data support the conclusions?

The manuscript must describe a technically sound piece of scientific research with data that supports the conclusions. Experiments must have been conducted rigorously, with appropriate controls, replication, and sample sizes. The conclusions must be drawn appropriately based on the data presented.

Reviewer #1: Yes

Reviewer #2: Yes

**********

2. Has the statistical analysis been performed appropriately and rigorously?

Reviewer #1: Yes

Reviewer #2: Yes

**********

3. Have the authors made all data underlying the findings in their manuscript fully available?

The PLOS Data policy requires authors to make all data underlying the findings described in their manuscript fully available without restriction, with rare exception (please refer to the Data Availability Statement in the manuscript PDF file). The data should be provided as part of the manuscript or its supporting information, or deposited to a public repository. For example, in addition to summary statistics, the data points behind means, medians and variance measures should be available. If there are restrictions on publicly sharing data—e.g. participant privacy or use of data from a third party—those must be specified.requires authors to make all data underlying the findings described in their manuscript fully available without restriction, with rare exception (please refer to the Data Availability Statement in the manuscript PDF file). The data should be provided as part of the manuscript or its supporting information, or deposited to a public repository. For example, in addition to summary statistics, the data points behind means, medians and variance measures should be available. If there are restrictions on publicly sharing data—e.g. participant privacy or use of data from a third party—those must be specified.

Reviewer #1: Yes

Reviewer #2: No

**********

4. Is the manuscript presented in an intelligible fashion and written in standard English?

PLOS ONE does not copyedit accepted manuscripts, so the language in submitted articles must be clear, correct, and unambiguous. Any typographical or grammatical errors should be corrected at revision, so please note any specific errors here.

Reviewer #1: Yes

Reviewer #2: Yes

**********

5. Review Comments to the Author

Please use the space provided to explain your answers to the questions above. You may also include additional comments for the author, including concerns about dual publication, research ethics, or publication ethics. (Please upload your review as an attachment if it exceeds 20,000 characters)

**Reviewer #1:**    I found this paper interesting to read and easy to follow. The experimental design and methods were well motivated and clearly explained. The statistical analysis is sound. The results were generally well presented.I found this paper interesting to read and easy to follow. The experimental design and methods were well motivated and clearly explained. The statistical analysis is sound. The results were generally well presented.

While my overall opinion on the paper leans positive, I have three main concerns, which I believe could at least be partly addressed in revisions:

1) QT is a television programme, where panellists give live answers to audience questions. This means that the real human responses that form the basis of this paper are transcribed from speech that occurred in a specific context. The LLM, on the other hand, generated text directly in a decontextualised setting, as provided by the system prompt (lines 84ff).

The authors acknowledge this discrepancy (e.g. line 443) but do not meaningfully engage with it. Fundamentally, human responses are not being evaluated in the setting and modality for which they were intended.

The paper would be stronger if it demonstrated that this discrepancy does not affect results. For example, human responses may include markers (such as references to other panellists names, disfluencies) that are perceived as inauthentic, incoherent, etc. when transcribed and decontextualised, but not when spoken on the programme. There may also be transcription errors, which LLMs would not have. This seems in scope for post-hoc analysis.

2) The paper finds that original content is different from impersonated content and concludes from this that AI may be used to generate targeted misinformation about the speaker’s point of view (lines 339ff). However, there is no analysis into *how* content differs in human vs LLM-impersonated content, and whether this difference does in fact correspond to “misrepresentation”. For this, it would be necessary to show that the LLM-impersonated answers differ from the human answers in their issue-specific stance.

It seems very plausible that LLMs follow different lines of argumentation but ultimately make similar points, which may (hypothetically) even be endorsed by the original speaker. Asking only about “difference in content”, which may be explained in many ways, cannot capture this. Consequently, the framing of the paper as relating to misinformation and deception seems only partly warranted.

3) The paper claims that human judgments are reliable but does not provide strong evidence for this. Agreement is modest at best (lines 359 following), and the supplementary variables show low agreement on several variables. Clearly, there is substantial noise in the data from subjective rating differences, which could be addressed by collecting and averaging across more ratings. If not, it would still be useful to be more open and specific about the (lack of) human agreement in the main body of the paper.

Minor notes:

- I found no mention of answer length in the paper. It seems very plausible that there is a difference in length between human and LLM answers, which may in turn affect at least some of the analyses. Some normalisation would likely be necessary?

- It may help the (non-UK) reader to note in the Data section that Question Time is a TV programme. The only mention of TV / television right now, I believe, is in the intro.

- On first reading, I was a bit confused by lines 117-119: “random speaker” may need brief explanation. Of course, this becomes clear later.

**Reviewer #2:**    The manuscript explores the capacity of large language models (LLMs) to impersonate public figures in political debates. The topic is timely and uses an interesting dataset. The paper is well written and methodologically ambitious. However, the strength of the conclusions is weakened by shortcomings in the experimental design and in the statistical analysis. The findings are interesting but should be interpreted with greater caution, given the limitations discussed below.The manuscript explores the capacity of large language models (LLMs) to impersonate public figures in political debates. The topic is timely and uses an interesting dataset. The paper is well written and methodologically ambitious. However, the strength of the conclusions is weakened by shortcomings in the experimental design and in the statistical analysis. The findings are interesting but should be interpreted with greater caution, given the limitations discussed below.

1. Experimental Design

The overall three-track design is conceptually sound allowing the author to assess perceptions under different conditions. Nonetheless, the construct of authenticity is insufficiently defined. It remains unclear whether participants interpreted it as authorship likelihood, realism, or plausibility, which may conflate distinct psychological dimensions. Moreover, each participant evaluated only a few items, which limits the reliability of individual responses. Besides, more details about the randomization of questions and speakers is required to ensure data is balanced. The manual inspection of generated responses lacks formal coding criteria or inter-rater checks, reducing transparency and reproducibility.

Suggestions for improvement:

Clarify the exact wording and intended meaning of each measure, particularly “authenticity.” Provide evidence that the randomization of stimuli was balanced across conditions. Include or reference inter-rater checks for the manual screening of generated responses.

2. Measures and Data Collection

The study relies entirely on self-reported Likert-scale judgments. This approach is valid for perceptual studies, but complementary behavioral or comprehension-based measures (e.g., response time, detection accuracy) would have strengthened construct validity. Some measures—especially “content similarity”—may overlap conceptually with “authenticity,” potentially introducing redundancy.

Suggestions for improvement:

Consider clarifying the independence of constructs and, if possible, provide an additional objective indicator of perceptual discrimination between real and impersonated responses. Provide information about possible moderator factors.

3. Statistical Analysis

The paper presents a detailed description of the statistical workflow and justifies the use of non-parametric tests. However, several inconsistencies remain. The analysis averages ordinal Likert data and reports means, standard deviations, and Cohen’s d, assuming interval properties. This is not strictly correct for ranked data and can inflate effect sizes. Inter-rater reliability (reported Cronbach’s α) indicates moderate agreement at best, suggesting considerable noise in the judgments. The treatment of each outcome variable (authenticity, coherence, relevance, content) as independent is also problematic, as they are conceptually and statistically correlated. Finally, while some corrections are applied, the overall analytical structure would benefit from mixed-effects or ordinal regression models that account for the nested and repeated nature of the data.

Suggestions for improvement:

Report medians and interquartile ranges alongside or instead of means. Use effect size measures suited for ordinal data (e.g., rank-biserial correlation or other relevant measure.) Reassess reliability using intraclass correlation or a mixed-model framework. Consider a multivariate or mixed-effects approach to handle correlated measures and participant/item dependencies.

4. Interpretation of Results

The finding that impersonated responses are perceived as more authentic, coherent, and relevant than the originals is statistically supported but likely overstated. Given the modest reliability and limited item sampling, the conclusion that participants “cannot discern” AI-generated content should be presented more cautiously. It is plausible that judgments reflect linguistic fluency rather than genuine attribution of authorship.

Suggestions for improvement:

Moderate the interpretive claims and emphasize the perceptual, rather than deceptive, nature of the results. Discuss alternative explanations such as stylistic consistency or linguistic polish in LLM outputs.

**********

6. PLOS authors have the option to publish the peer review history of their article (what does this mean?). If published, this will include your full peer review and any attached files.). If published, this will include your full peer review and any attached files.

If you choose “no”, your identity will remain anonymous but your review may still be made public.

**Do you want your identity to be public for this peer review?**    For information about this choice, including consent withdrawal, please see our For information about this choice, including consent withdrawal, please see our Privacy Policy..

Reviewer #1: No

Reviewer #2: No

**********

[NOTE: If reviewer comments were submitted as an attachment file, they will be attached to this email and accessible via the submission site. Please log into your account, locate the manuscript record, and check for the action link "View Attachments". If this link does not appear, there are no attachment files.]

While revising your submission, please upload your figure files to the Preflight Analysis and Conversion Engine (PACE) digital diagnostic tool, https://pacev2.apexcovantage.com/. PACE helps ensure that figures meet PLOS requirements. To use PACE, you must first register as a user. Registration is free. Then, login and navigate to the UPLOAD tab, where you will find detailed instructions on how to use the tool. If you encounter any issues or have any questions when using PACE, please email PLOS at . PACE helps ensure that figures meet PLOS requirements. To use PACE, you must first register as a user. Registration is free. Then, login and navigate to the UPLOAD tab, where you will find detailed instructions on how to use the tool. If you encounter any issues or have any questions when using PACE, please email PLOS at figures@plos.org. Please note that Supporting Information files do not need this step.. Please note that Supporting Information files do not need this step.

# Author comment: Author response to Decision Letter 1

**DOI:** 10.1371/journal.pone.0347757.r002

25 Nov 2025

Response to Reviews

We thank the two reviewers, as well as the editor, for their feedback. Based on the comments, we prepared a revision of our manuscript for your consideration. To aid the reviewers, all changes are marked in blue within the updated manuscript.

Editor

Comment E.1: Reviewer 1 comments the design and analysis but raises concerns about the contextual asymmetry between the original human responses and the LLM-generated ones. Since the human content was transcribed from a live television programme (“Question Time”), it may include disfluencies, references, or transcription artifacts that affect perceptions differently than the directly generated LLM text. The reviewer suggests adding a post-hoc robustness check or qualitative analysis to demonstrate that these contextual or transcription-related differences do not drive the main effects.

Response E.1: We address this issue by conducting additional checks such as automated spell checking and a manual analysis of the responses to rule out such effects. The additional results are reported in the new supplemental material S3.

For more details, we refer to Response R1.1.

Comment E.2: Relatedly, Reviewer 1 encourages you to expand the discussion of content differences between human and impersonated responses. Specifically, the paper would benefit from showing how these differ substantively; for example, by analyzing whether impersonations diverge in stance or argumentation, rather than merely in surface-level content. This would clarify whether the framing of “misrepresentation” is warranted.

Response E.2: Based on these understandable concerns, we conduct additional manual analysis, in which we compare the stance conveyed by the actual and the generated response. While there are indeed cases in which the differences are rather in how a stance is conveyed, we also find a non-negligible number (26%) of the samples that we checked, where the differences in content were indeed differences in the stance that was conveyed.

For more details, we refer to Response 1.2.

Comment E.3: Finally, the reviewer notes that the reliability of human ratings is modest and recommends greater transparency about inter-rater variability, or, if feasible, additional averaging or collection of ratings to reduce subjective noise.

Response E.3: While we acknowledge this concern, we want to highlight that the differences in the judgments are neither random (i.e., completely unreliable) nor polar (i.e., opposite judgments), but rather on neighboring items on the Likert scale. We discuss these concerns in detail in the “Human judgment is reliable” section, which dedicates a whole paragraph to this issue. While we acknowledge that additional data collection could possibly help, we do not believe that there will be substantial changes, given our already very large sample size of 520 question/response pairs.

For more details, we refer to Response 1.3.

Comment E.4: Minor notes include adding a brief explanation that Question Time is a television programme, clarifying “random speaker” in the methods, and checking for potential answer length differences that could influence results.

Response E.4: We now address all these concerns explicitly in the paper, including an additional analysis on the random speaker assignments and the influence of answer lengths.

For more details, we refer to Responses 1.4, 1.5, and 1.6.

Comment E.5: 2. Conceptual and methodological refinements (Reviewer 2)

Reviewer 2 finds the study compelling but identifies several areas where methodological clarification is needed:

Construct clarity: The meaning of authenticity should be defined more precisely, whether it captures authorship likelihood, realism, or plausibility. Reviewers recommend clarifying how this construct differs from related ones (e.g., coherence or content similarity) and ensuring that participants understood it consistently.

Response E.5: We clarify our definition for authenticity as requested.

For more details, refer to Response 2.1.

Comment E.6: Experimental design: Provide more detail on the randomization and balancing of stimuli across conditions. If possible, include or reference any inter-rater checks used during the manual screening of generated responses.

Response E.6: We acknowledge that we should have been clearer regarding these issues. We almost always use the full data set and avoid sampling. The only case where we sample is the random assignment of speakers, as well as the new qualitative analysis of answers we already discussed above. We provide additional analysis for this aspect. We also failed to clarify why no inter-rater agreements are reported for our own manual analysis: all cases in which the second rater disagreed with the original judgment by the first rater were discussed and agreement was reached in all cases.

For more details, we refer to Responses 2.3 and 2.4.

Comment E.7: Measurement approach: Consider complementing subjective Likert judgments with an objective or behavioral indicator (e.g., response times or accuracy), or at least acknowledge the limitations of relying solely on self-reported data.

Response E.7: While this would yield insightful additional data, such data cannot be reliably obtained with the only survey method we used. While there is possibly an impact because of unobserved confounding effects, this is highly unlikely given our sample size. Still, we acknowledge this limitation in the “Human judgments are reliable” section.

For more details we refer to Response 2.5.

Comment E.8: Statistical analysis: The reviewer suggests using non-parametric or mixed-effects models better suited to ordinal and nested data, reporting medians and interquartile ranges, and revising effect size measures accordingly. You might also re-evaluate inter-rater reliability using intraclass correlation coefficients.

Response E.8: We carefully considered all suggestions by the reviewer and partially updated our methods. We revised the effect sizes to be non-parametric (almost no changes, one increase in in effect strength). All non-parametric markers are now reported next to the parametric mean and standard deviation in the appendix and we explain why we kept the mean and standard deviation in the main body of the paper. We did not switch to a different statistical model (i.e., no variant of a linear model), because we directly want to observe how the variables change and not how the combination of variables explains a change in dependent variable.

For more details, we refer to Response 2.6.

Comment E.9: Interpretation: Finally, temper the strength of the claims about deception or indistinguishability between AI and human responses. Reviewer 2 encourages reframing your findings as perceptual rather than deceptive effects, potentially driven by linguistic fluency or stylistic uniformity.

Response E.9: While our additional analysis further supports our results, we acknowledge that some wordings were too strong in the initial submission as our results are limited to the perception of our participants. We consequently edited the wording in key locations of the paper such as the abstract and the results section.

For more details, we refer to Response 2.7.

Journal Requirements:

Comment J.1: When submitting your revision, we need you to address these additional requirements.

Please ensure that your manuscript meets PLOS ONE's style requirements, including those for file naming. The PLOS ONE style templates can be found at

https://journals.plos.org/plosone/s/file?id=wjVg/PLOSOne_formatting_sample_main_body.pdf and

https://journals.plos.org/plosone/s/file?id=ba62/PLOSOne_formatting_sample_title_authors_affiliations.pdf

Response J.1: We updated all file names and types to match the requirements.

Comment J.2: Please update your submission to use the PLOS LaTeX template. The template and more information on our requirements for LaTeX submissions can be found at http://journals.plos.org/plosone/s/latex.

Response J.2: We updated our submission to use the latest version of the LaTeX style file (Version 3.7 from Aug 2025)

Comment J.3: We note that the grant information you provided in the ‘Funding Information’ and ‘Financial Disclosure’ sections do not match.

When you resubmit, please ensure that you provide the correct grant numbers for the awards you received for your study in the ‘Funding Information’ section.

Response J.3: Thank you for reporting this. We now clarify the matter in the cover letter.

Comment J.4: Thank you for stating the following financial disclosure:

A.H. work was partially funded by the VolkswagenStiftung under grant Az. 98544 ‘Deliberation Laboratory’

URL: https://www.volkswagenstiftung.de/

The funders had no role in the study whatsoever.

Please state what role the funders took in the study. If the funders had no role, please state: "The funders had no role in study design, data collection and analysis, decision to publish, or preparation of the manuscript."

If this statement is not correct you must amend it as needed.

Please include this amended Role of Funder statement in your cover letter; we will change the online submission form on your behalf.

Response J.4: The funders had no role in the study and we clarify this in the cover letter.

Comment J.5: Thank you for stating the following in the Acknowledgments Section of your manuscript:

The work reported on in this paper was partially funded by the VolkswagenStiftung under grant Az. 98544 ‘Deliberation Laboratory’

We note that you have provided funding information that is not currently declared in your Funding Statement. However, funding information should not appear in the Acknowledgments section or other areas of your manuscript. We will only publish funding information present in the Funding Statement section of the online submission form.

Please remove any funding-related text from the manuscript and let us know how you would like to update your Funding Statement. Currently, your Funding Statement reads as follows:

A.H. work was partially funded by the VolkswagenStiftung under grant Az. 98544 ‘Deliberation Laboratory’

URL: https://www.volkswagenstiftung.de/

The funders had no role in the study whatsoever.

Please include your amended statements within your cover letter; we will change the online submission form on your behalf.

Response J.5: Thank you for this clarification. We provide the amended statements in our cover letter and drop the acknowledgements section from the manuscript.

Comment J.6: Thank you for uploading your study's underlying data set. Unfortunately, the repository you have noted in your Data Availability statement does not qualify as an acceptable data repository according to PLOS's standards.

At this time, please upload the minimal data set necessary to replicate your study's findings to a stable, public repository (such as figshare or Dryad) and provide us with the relevant URLs, DOIs, or accession numbers that may be used to access these data. For a list of recommended repositories and additional information on PLOS standards for data deposition, please see https://journals.plos.org/plosone/s/recommended-repositories.

Response J.6: We noticed that the DOI-reference to our long-term archive at Zenodo was missing the last character. This is now fixed and the new Data Availability statement is:

“All materials are available online in the form of a replication package that contains the data: https://doi.org/10.5281/zenodo.12698364”

Comment J.7: Please include your full ethics statement in the ‘Methods’ section of your manuscript file. In your statement, please include the full name of the IRB or ethics committee who approved or waived your study, as well as whether or not you obtained informed written or verbal consent. If consent was waived for your study, please include this information in your statement as well.

Response J.7: We now have an additional subsection at the end of the Methods section with the full ethics statement. We extend the ethics statement to clarify that the obtained consent was written.

Comment J.8: If the reviewer comments include a recommendation to cite specific previously published works, please review and evaluate these publications to determine whether they are relevant and should be cited. There is no requirement to cite these works unless the editor has indicated otherwise.

Response J.8: Thank you for this comment. There were no recommendations for additional references in the reviews.

Reviewer 1

Comment R1.1: QT is a television programme, where panellists give live answers to audience questions. This means that the real human responses that form the basis of this paper are transcribed from speech that occurred in a specific context. The LLM, on the other hand, generated text directly in a decontextualised setting, as provided by the system prompt (lines 84ff).

The authors acknowledge this discrepancy (e.g. line 443) but do not meaningfully engage with it. Fundamentally, human responses are not being evaluated in the setting and modality for which they were intended.

The paper would be stronger if it demonstrated that this discrepancy does not affect results. For example, human responses may include markers (such as references to other panellists names, disfluencies) that are perceived as inauthentic, incoherent, etc. when transcribed and decontextualised, but not when spoken on the programme. There may also be transcription errors, which LLMs would not have. This seems in scope for post-hoc analysis.

Response R1.1: This is a good point and would be a viable alternative explanation for our results. We add additional analysis regarding this (and other) alternative hypothesis to the supplemental material in Appendix S3. We decided to exclude this from the main body of the paper in order to keep the presentation crisp and only add a reference to this new supplemental material to the end of the Section “Statistical analysis”. In summary, errors have no correlation with judgments. While transcriptions have more errors (2.4 on average vs. 0.26 for generated responses), most errors are minor, e.g., missing commas between clauses of compound sentences. In 10% of the original responses, we manually checked if actual responses contain references to other panelists and we do not find such cases. We add this information in Section “Original Debate Content.”

Comment R1.2: The paper finds that original content is different from impersonated content and concludes from this that AI may be used to generate targeted misinformation about the speaker’s point of view (lines 339ff). However, there is no analysis into *how* content differs in human vs LLM-impersonated content, and whether this difference does in fact correspond to “misrepresentation”. For this, it would be necessary to show that the LLM-impersonated answers differ from the human answers in their issue-specific stance.

It seems very plausible that LLMs follow different lines of argumentation but ultimately make similar points, which may (hypothetically) even be endorsed by the original speaker. Asking only about “difference in content”, which may be explained in many ways, cannot capture this. Consequently, the framing of the paper as relating to misinformation and deception seems only partly warranted.

Response R1.2: Thank you for this comment. We acknowledge that our definition of the term “content” as “difference in overall meaning” is too broad to directly justify these statements. To mitigate this, we include additional qualitative analysis on the share of data that was judged as different in content, indicated by a negative mean value of the “content” judgments by the study participants. The goal of this qualitative analysis is to establish whether the differences in content are indeed about misrepresentation. For this, we focus on the stance, i.e., if the response arrives at the same conclusion, argues for the same points or takes the same stance. This focus excludes the use of rhetorical devices as other means to convey a point, which may be used to make misrepresentations authentic. Additionally, we check if the respons

Attachment Submitted filename: PLOS-One-QT-GPT-Response.pdf

# Peer review: Decision Letter 1

**DOI:** 10.1371/journal.pone.0347757.r003

**Authors:** Mohammad Salah Hassan

23 Mar 2026

PONE-D-25-15257R1

LLM-impersonated debate contributions are more authentic, relevant and coherent than their original: A representative study using BBC1’s Question Time

PLOS One

Dear Dr. Herbold,

Thank you for submitting your manuscript to PLOS ONE. After careful consideration, we feel that it has merit but does not fully meet PLOS ONE’s publication criteria as it currently stands. Therefore, we invite you to submit a revised version of the manuscript that addresses the points raised during the review process.

Please submit your revised manuscript by May 07 2026 11:59PM. If you will need more time than this to complete your revisions, please reply to this message or contact the journal office at plosone@plos.org. When you're ready to submit your revision, log on to https://www.editorialmanager.com/pone/ and select the 'Submissions Needing Revision' folder to locate your manuscript file.. When you're ready to submit your revision, log on to https://www.editorialmanager.com/pone/ and select the 'Submissions Needing Revision' folder to locate your manuscript file.

Please include the following items when submitting your revised manuscript:

- A letter that responds to each point raised by the academic editor and reviewer(s). You should upload this letter as a separate file labeled 'Response to Reviewers'.
- A marked-up copy of your manuscript that highlights changes made to the original version. You should upload this as a separate file labeled 'Revised Manuscript with Track Changes'.
- An unmarked version of your revised paper without tracked changes. You should upload this as a separate file labeled 'Manuscript'.

-->If you would like to make changes to your financial disclosure, please include your updated statement in your cover letter. Guidelines for resubmitting your figure files are available below the reviewer comments at the end of this letter.

If applicable, we recommend that you deposit your laboratory protocols in protocols.io to enhance the reproducibility of your results. Protocols.io assigns your protocol its own identifier (DOI) so that it can be cited independently in the future. For instructions see: https://journals.plos.org/plosone/s/submission-guidelines#loc-laboratory-protocols. Additionally, PLOS ONE offers an option for publishing peer-reviewed Lab Protocol articles, which describe protocols hosted on protocols.io. Read more information on sharing protocols at . Additionally, PLOS ONE offers an option for publishing peer-reviewed Lab Protocol articles, which describe protocols hosted on protocols.io. Read more information on sharing protocols at https://plos.org/protocols?utm_medium=editorial-email&utm_source=authorletters&utm_campaign=protocols..

We look forward to receiving your revised manuscript.

Kind regards,

Mohammad Salah Hassan, Ph.D

Academic Editor

PLOS One

Journal Requirements:

1. If the reviewer comments include a recommendation to cite specific previously published works, please review and evaluate these publications to determine whether they are relevant and should be cited. There is no requirement to cite these works unless the editor has indicated otherwise.

2. Please review your reference list to ensure that it is complete and correct. If you have cited papers that have been retracted, please include the rationale for doing so in the manuscript text, or remove these references and replace them with relevant current references. Any changes to the reference list should be mentioned in the rebuttal letter that accompanies your revised manuscript. If you need to cite a retracted article, indicate the article’s retracted status in the References list and also include a citation and full reference for the retraction notice.

Additional Editor Comments :

Dear Authors,

The revised manuscript, PONE-D-25-15257R1, entitled “LLM-impersonated debate contributions are more authentic, relevant and coherent than their original: A representative study using BBC1’s Question Time,” has now been reviewed.

Please accept my sincere apologies for the delay in reaching this decision, as one of the invited reviewers did not reply and additional time was therefore needed to secure a further review.

Reviewer 1 recommends acceptance and confirms that the authors have adequately addressed the comments raised in the previous round. The reviewer considers the manuscript technically sound, the statistical analysis appropriate, the data availability satisfactory, and the language clear and intelligible.

A few very minor points remain, but these should be treated as final editorial clean-up rather than substantive revisions. In line with the reviewer’s observations, the authors should clarify the limitation concerning the mismatch in context and modality between the original human responses and the LLM-generated responses, and moderate the “threat” framing where the wording may still appear stronger than warranted. In addition, a few minor editorial inconsistencies should be corrected in the final files, including residual typographical and spacing issues, consistency of the manuscript title across all submitted documents, and the final Data Availability link/DOI.

These minor matters do not affect the overall positive recommendation. Based on the reviewer’s report and my own assessment, I recommend acceptance of the manuscript subject to final minor editorial polishing.

Kind regards,

Mohammed Salah Alazzawi, PhD

[Note: HTML markup is below. Please do not edit.]

Reviewers' comments:

Reviewer's Responses to Questions

**Comments to the Author**

1. If the authors have adequately addressed your comments raised in a previous round of review and you feel that this manuscript is now acceptable for publication, you may indicate that here to bypass the “Comments to the Author” section, enter your conflict of interest statement in the “Confidential to Editor” section, and submit your "Accept" recommendation.

Reviewer #1: All comments have been addressed

Reviewer #3: All comments have been addressed

**********

2. Is the manuscript technically sound, and do the data support the conclusions?

The manuscript must describe a technically sound piece of scientific research with data that supports the conclusions. Experiments must have been conducted rigorously, with appropriate controls, replication, and sample sizes. The conclusions must be drawn appropriately based on the data presented.

Reviewer #1: Yes

Reviewer #3: Yes

**********

3. Has the statistical analysis been performed appropriately and rigorously?

Reviewer #1: Yes

Reviewer #3: Yes

**********

4. Have the authors made all data underlying the findings in their manuscript fully available?

The PLOS Data policy requires authors to make all data underlying the findings described in their manuscript fully available without restriction, with rare exception (please refer to the Data Availability Statement in the manuscript PDF file). The data should be provided as part of the manuscript or its supporting information, or deposited to a public repository. For example, in addition to summary statistics, the data points behind means, medians and variance measures should be available. If there are restrictions on publicly sharing data—e.g. participant privacy or use of data from a third party—those must be specified.requires authors to make all data underlying the findings described in their manuscript fully available without restriction, with rare exception (please refer to the Data Availability Statement in the manuscript PDF file). The data should be provided as part of the manuscript or its supporting information, or deposited to a public repository. For example, in addition to summary statistics, the data points behind means, medians and variance measures should be available. If there are restrictions on publicly sharing data—e.g. participant privacy or use of data from a third party—those must be specified.

Reviewer #1: Yes

Reviewer #3: Yes

**********

5. Is the manuscript presented in an intelligible fashion and written in standard English?

PLOS ONE does not copyedit accepted manuscripts, so the language in submitted articles must be clear, correct, and unambiguous. Any typographical or grammatical errors should be corrected at revision, so please note any specific errors here.

Reviewer #1: Yes

Reviewer #3: Yes

**********

6. Review Comments to the Author

Please use the space provided to explain your answers to the questions above. You may also include additional comments for the author, including concerns about dual publication, research ethics, or publication ethics. (Please upload your review as an attachment if it exceeds 20,000 characters)

Reviewer #1: I thank the authors for their detailed response to my first review. My concerns have largely been addressed, and I am happy to recommend acceptance. However, I would encourage the authors to engage with the following points:

1) I still believe that the mismatch in context and modality between the original human responses and the LLM-generated responses cannot be ruled out as at least a partial explanation of results. Length and prevalence of spelling errors may not individually have strong correlations with perceptions of authenticity etc., but their combination (and other unobserved factors) may still partially explain human judgments. A true like-for-like comparison would compare human-written textual responses to LLM-written responses. Since this comparison cannot be made, I think this should be clearly acknowledged as a limitation in the main body discussion.

2) I would encourage the authors to be clearer in their “threat model”: What exactly is the new threat created by LLM impersonation? And to what extent do the experiments in this paper measure this new threat? For example, I would argue that not all “made-up content” is equally problematic – LLM impersonation poses a threat only if the LLM-generated content *misrepresents* the speaker’s views *while still being perceived as authentic*. This is not something the paper tests for! The small qualitative analysis shows that there are 13 of 50 cases where the LLM-generated response misaligns in stance, but this is not enough to make claims about general LLM tendency to misrepresent, or how this misrepresentation impacts authenticity judgments. Personally, I would therefore tone down the “threat” language throughout the paper and simply provide a sober description of results related to authenticity, and pointers to open questions for future work.

Minor notes:

- line 18: typo and missing whitespace

- line 184: missing whitespace

Reviewer #3: This paper has been significantly improved. Thank you very much for your efforts in making the manuscript stronger and more polished after the revision.

**********

7. PLOS authors have the option to publish the peer review history of their article (what does this mean?). If published, this will include your full peer review and any attached files.). If published, this will include your full peer review and any attached files.

If you choose “no”, your identity will remain anonymous but your review may still be made public.

**Do you want your identity to be public for this peer review?**    For information about this choice, including consent withdrawal, please see our For information about this choice, including consent withdrawal, please see our Privacy Policy..

Reviewer #1: No

Reviewer #3: No

**********

[NOTE: If reviewer comments were submitted as an attachment file, they will be attached to this email and accessible via the submission site. Please log into your account, locate the manuscript record, and check for the action link "View Attachments". If this link does not appear, there are no attachment files.]

To ensure your figures meet our technical requirements, please review our figure guidelines: https://journals.plos.org/plosone/s/figures

You may also use PLOS’s free figure tool, NAAS, to help you prepare publication quality figures: https://journals.plos.org/plosone/s/figures#loc-tools-for-figure-preparation.

NAAS will assess whether your figures meet our technical requirements by comparing each figure against our figure specifications.

# Author comment: Author response to Decision Letter 2

**DOI:** 10.1371/journal.pone.0347757.r004

25 Mar 2026

We fixed all reported minor issues. For the two comments from R1, we added the following to the main body of the manuscript in the Section "Discussion and conclusion" (1) to highlight the limitation due to possible unobservable factors affecting our results and (2) to frame the generalizability more carefully:

(1) "While we rule out length and grammatical errors as possible sources for differences in authenticity, we cannot rule out that there are unobserved factors introduced by our experiment design."

(2) "We note that our setting did not study this targeted misinformation, i.e., we did not prescribe the position the LLM should express. Future work needs to study if LLMs are still perceived as authentic when used in such a targeted manner."

# Editor report: Decision Letter 2

**DOI:** 10.1371/journal.pone.0347757.r005

**Authors:** Mohammad Salah Hassan

7 Apr 2026

LLM-impersonated debate contributions are more authentic, relevant and coherent than their original: A representative study using BBC1’s Question Time

PONE-D-25-15257R2

Dear Dr. Authors,

We’re pleased to inform you that your manuscript has been judged scientifically suitable for publication and will be formally accepted for publication once it meets all outstanding technical requirements.

Within one week, you’ll receive an e-mail detailing the required amendments. When these have been addressed, you’ll receive a formal acceptance letter and your manuscript will be scheduled for publication.

An invoice will be generated when your article is formally accepted. Please note, if your institution has a publishing partnership with PLOS and your article meets the relevant criteria, all or part of your publication costs will be covered. Please make sure your user information is up-to-date by logging into Editorial Manager at Editorial Manager® and clicking the ‘Update My Information' link at the top of the page. For questions related to billing, please contact  and clicking the ‘Update My Information' link at the top of the page. For questions related to billing, please contact billing support..

If your institution or institutions have a press office, please notify them about your upcoming paper to help maximize its impact. If they’ll be preparing press materials, please inform our press team as soon as possible -- no later than 48 hours after receiving the formal acceptance. Your manuscript will remain under strict press embargo until 2 pm Eastern Time on the date of publication. For more information, please contact onepress@plos.org.

Kind regards,

Mohammad Salah Hassan, Ph.D

Academic Editor

PLOS One

Additional Editor Comments (optional):

Dear Authors,

Thank you for submitting the revised version of your manuscript, “LLM-impersonated debate contributions are more authentic, relevant and coherent than their original: A representative study using BBC1’s Question Time.”

The manuscript has improved substantially, and the concerns raised in the previous round have been adequately addressed.

I am pleased to inform you that I recommend acceptance of the manuscript for publication in PLOS ONE.

Kind regards,

Reviewers' comments:

# Editor report: Acceptance letter

**DOI:** 10.1371/journal.pone.0347757.r006

**Authors:** Mohammad Salah Hassan

PONE-D-25-15257R2

PLOS One

Dear Dr. Herbold,

I'm pleased to inform you that your manuscript has been deemed suitable for publication in PLOS One. Congratulations! Your manuscript is now being handed over to our production team.

At this stage, our production department will prepare your paper for publication. This includes ensuring the following:

* All references, tables, and figures are properly cited

* All relevant supporting information is included in the manuscript submission,

* There are no issues that prevent the paper from being properly typeset

You will receive further instructions from the production team, including instructions on how to review your proof when it is ready. Please keep in mind that we are working through a large volume of accepted articles, so please give us a few days to review your paper and let you know the next and final steps.

Lastly, if your institution or institutions have a press office, please let them know about your upcoming paper now to help maximize its impact. If they'll be preparing press materials, please inform our press team within the next 48 hours. Your manuscript will remain under strict press embargo until 2 pm Eastern Time on the date of publication. For more information, please contact onepress@plos.org.

You will receive an invoice from PLOS for your publication fee after your manuscript has reached the completed accept phase. If you receive an email requesting payment before acceptance or for any other service, this may be a phishing scheme. Learn how to identify phishing emails and protect your accounts at https://explore.plos.org/phishing.

If we can help with anything else, please email us at customercare@plos.org.

Thank you for submitting your work to PLOS ONE and supporting open access.

Kind regards,

PLOS ONE Editorial Office Staff

on behalf of

Dr. Mohammad Salah Hassan

Academic Editor

PLOS One
